# Supplementary material for: Architecture of symbiotic dinoflagellate photosystem I–light-harvesting supercomplex in Symbiodinium
Source: Nat Commun. 2024 Mar 16;15:2392. doi: 10.1038/s41467-024-46791-x (PMC10944487; doi:10.1038/s41467-024-46791-x)
Supplement: Supplementary file 1 — Supplementary Information [file 41467_2024_46791_MOESM1_ESM.pdf]

Supplementary Information

for

Zhao et al., Architecture of symbiotic dinoflagellate photosystem I–light-harvesting supercomplex in *Symbiodinium*

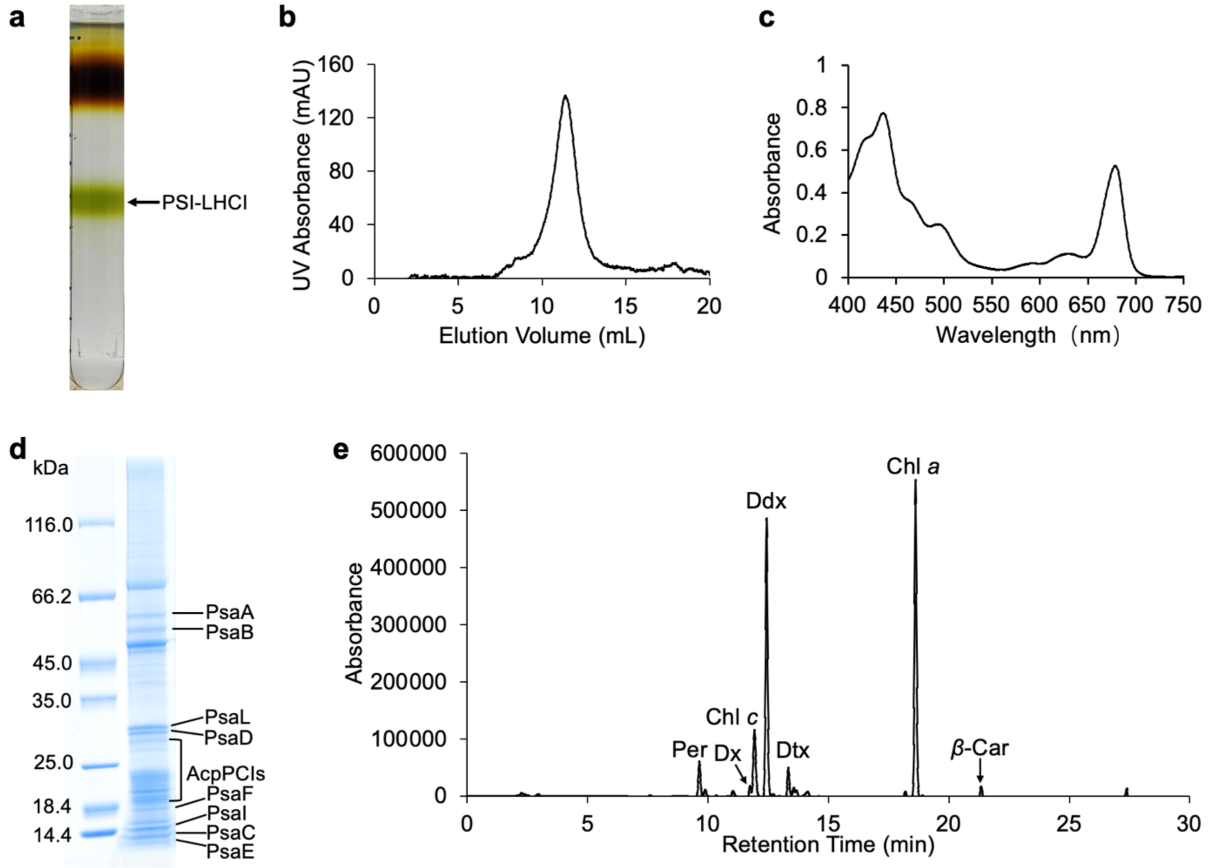

Supplementary Fig. 1. Preparation and characterization of PSI–AcpPCI from *Symbiodinium sp*. a, Isolation of the PSI–AcpPCI supercomplex by ultracentrifugation using sucrose density gradient. **b**, Purification of the PSI–AcpPCI supercomplex by size-exclusion chromatography. **c**, Room-temperature absorption spectra of the PSI–AcpPCI supercomplex. **d**, SDS-PAGE analysis of the PSI–AcpPCI supercomplex. The protein composition of the bands was indicated based on mass spectrometry analysis (Supplementary Data 1). **e**, Analysis of the pigment composition of PSI–AcpPCI by HPLC, recorded at 445 nm. Based on the characteristic absorption spectrum of each peak fraction, six major pigment peaks were identified as peridinin (Per), dinoxanthin (Dx), chlorophyll *c* (Chl *c*), diadinoxanthin (Ddx), diatoxanthin (Dtx), chlorophyll *a* (Chl *a*) and *β*-carotene (*β*-Car) respectively. These experiments were performed for more than five times, and the same results were obtained reproducibly. Source data for Supplementary Figs. 1b, 1c, and 1e are provided.

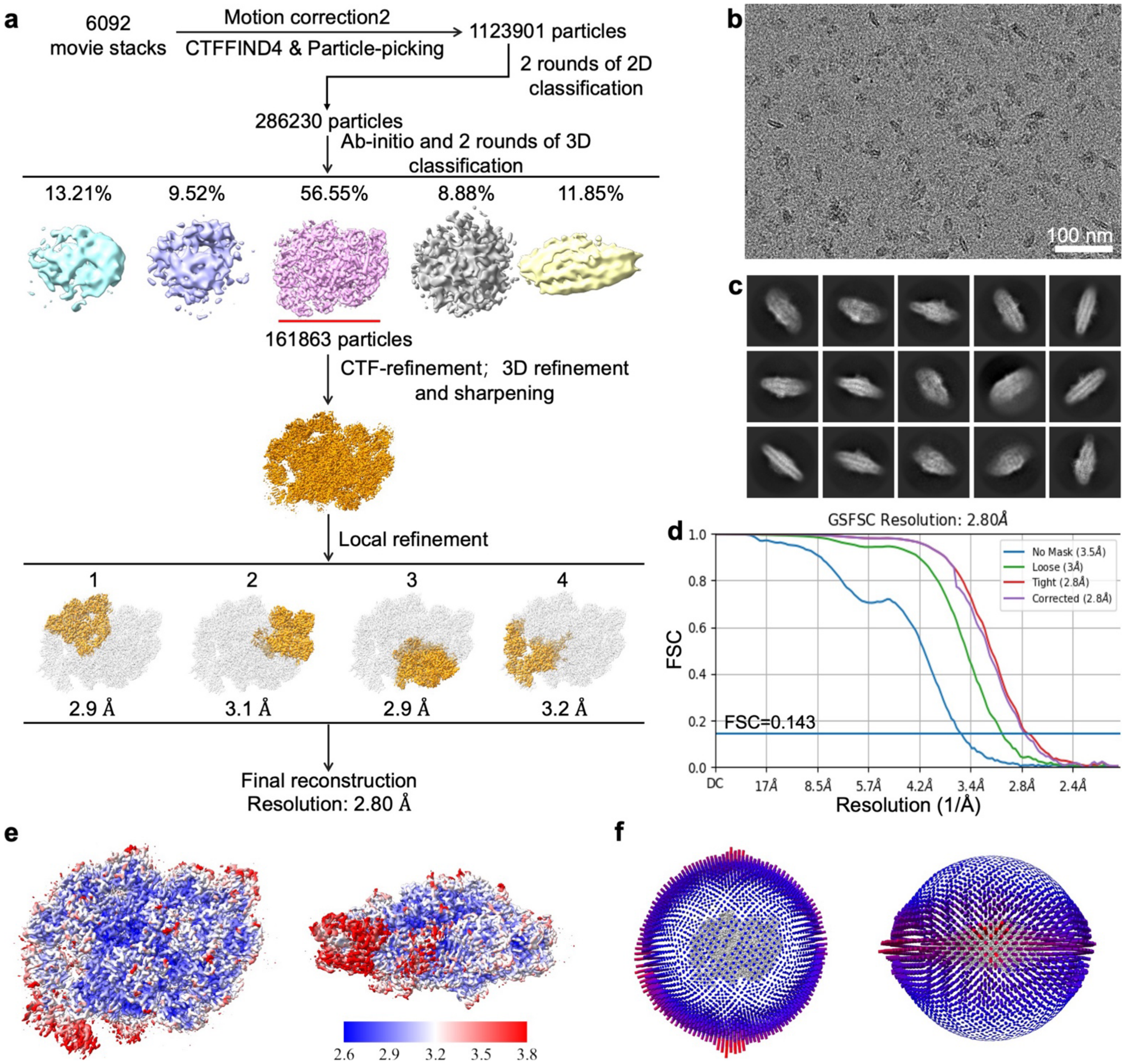

Supplementary Fig. 2. Cryo-EM data collection and processing of the PSI–AcpPCI supercomplex and evaluation of the cryo-EM map quality. **a**, Schematic flowchart for the cryo-EM data processing. **b**, A representative cryo-EM micrograph of the PSI–AcpPCI supercomplex. **c**, Representative 2D classes of the PSI–AcpPCI supercomplex. The box size is 396 Å. **d**, The gold standard Fourier shell correlation (FSC) curves for estimation of the resolution of the density map with criterion of 0.143. **e**, Local resolution distributions of the cryo-EM map estimated by ResMap. **f**, Angular distribution of particles used for reconstruction of the final density map.

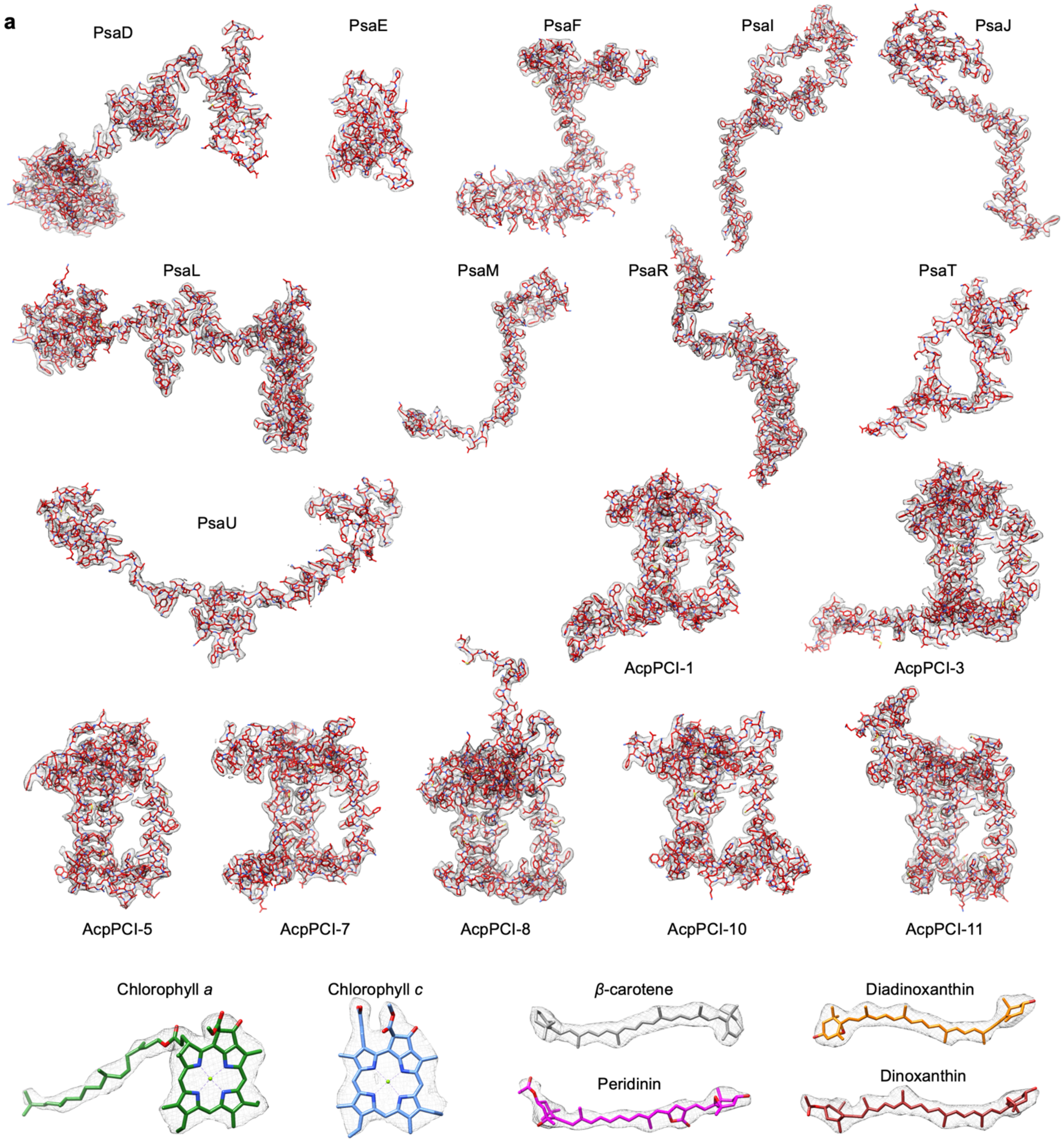
a
PsaD
PsaE
PsaF
PsaI
PsaJ
PsaL
PsaM
PsaR
PsaT
PsaU
AcpPCI-1
AcpPCI-3
AcpPCI-5
AcpPCI-7
AcpPCI-8
AcpPCI-10
AcpPCI-11
Chlorophyll *a*
Chlorophyll *c*
β-carotene
Diadinoxanthin
Peridinin
Dinoxanthin

b

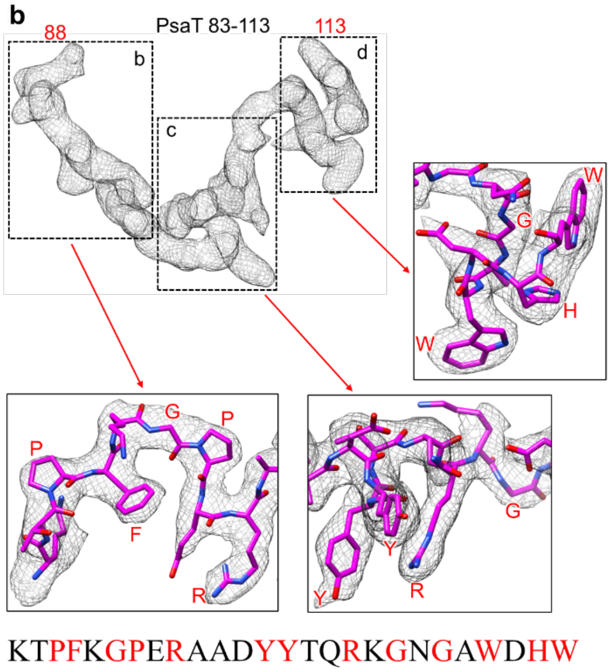

c

Speculation sequence 1 (88-113)	SLADSSLPGATRAGAYYTDRIAIGAWAQW
**Blastp sequence 1-1**	**KTPFKGPERAADYYTQRKGNGAWDHW**
Blastp sequence 1-2	ADSSCPPPPRAASPWRDRSPCHRWSR
Speculation sequence 2 (88-113)	TLADASLAGATRAGAYFTIRVAIGAWAQW
Blastp sequence 2-1	LAVTSRPPAYFTLRRPCGVASRW
Speculation sequence 3 (88-113)	SLADSAQPGATRAGAYFTERVAIGAWAQW
Blastp sequence 3-1	SSAPSARPGKKRAGEYFDCRKA
Blastp sequence 3-2	GPERAADYYTQRKGNGAWDHW

d

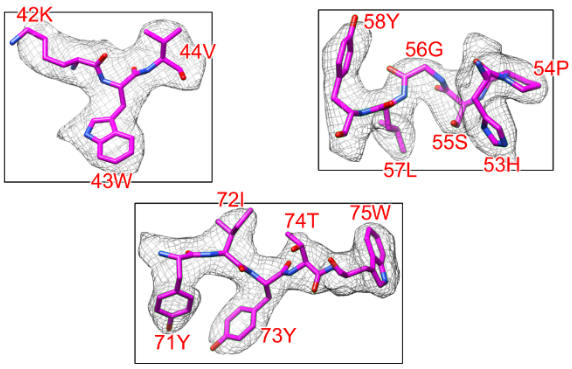

e

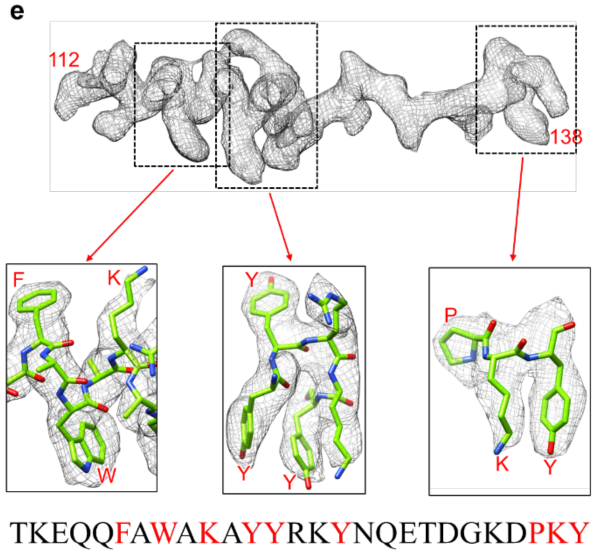

f

Speculation sequence 1 (112-138)	TPADMFAWSKAYHYMYQIDATGRTPKW
**Blastp sequence 1-1**	**TKEQQFAWAKAYYRKYNQETDGKDPKY**
Blastp sequence 1-2	SRHYHGIFYCDCTGRSPE
Blastp sequence 1-3	ADGFAWPEALHLFGQVD
Speculation sequence 2 (112-138)	TPADEFAQSKAYFYQFQIDATGRTPKW
Blastp sequence 2-1	PADQGAEVQAHLCDLLLSRTGRKP
Speculation sequence 3 (112-138)	TPADKFARSKAYHYMYQIDATGRTPKW
Blastp sequence 3-1	PWCKHARSRTSSEMKSLAPSGRTPRW
Blastp sequence 3-2	PDDSFARSKAQLCMGKMGKTGKIGK

g

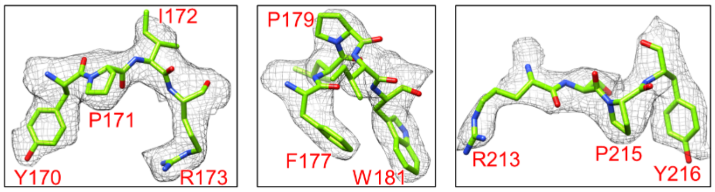

Supplementary Fig. 3. Cryo-EM density maps of protein subunits and representative cofactors in the PSI–AcpPCI supercomplex of *Symbiodinium* and the sequences identification of PsaT and PsaU. a, Cryo-EM density maps and structures of the PsaT subunits, PsaU subunits, the core subunits and AcpPCIs with extended terminal domains, and pigment molecules. **b, e**, Selected regions of the density maps of PsaT (**b**) and PsaU (**e**) with higher resolution (upper panel), and fitting of the proper sequence from the candidates shown in **c** and **f** into the selected region (bottom panel). Distinguishable amino acids are colored in red and labeled. **c, f**, Sequences speculated based on the selected map regions of PsaT (**c**) and PsaU (**f**) (Speculation sequence) and potential sequences (Blastp sequence) identified from the transcriptome sequences through blastp using the speculation sequences. The proper sequences in **b** and **e** are bold. **d, g**, The suitability of the proper sequence with the density map in other regions. Distinguishable amino acids are labeled.

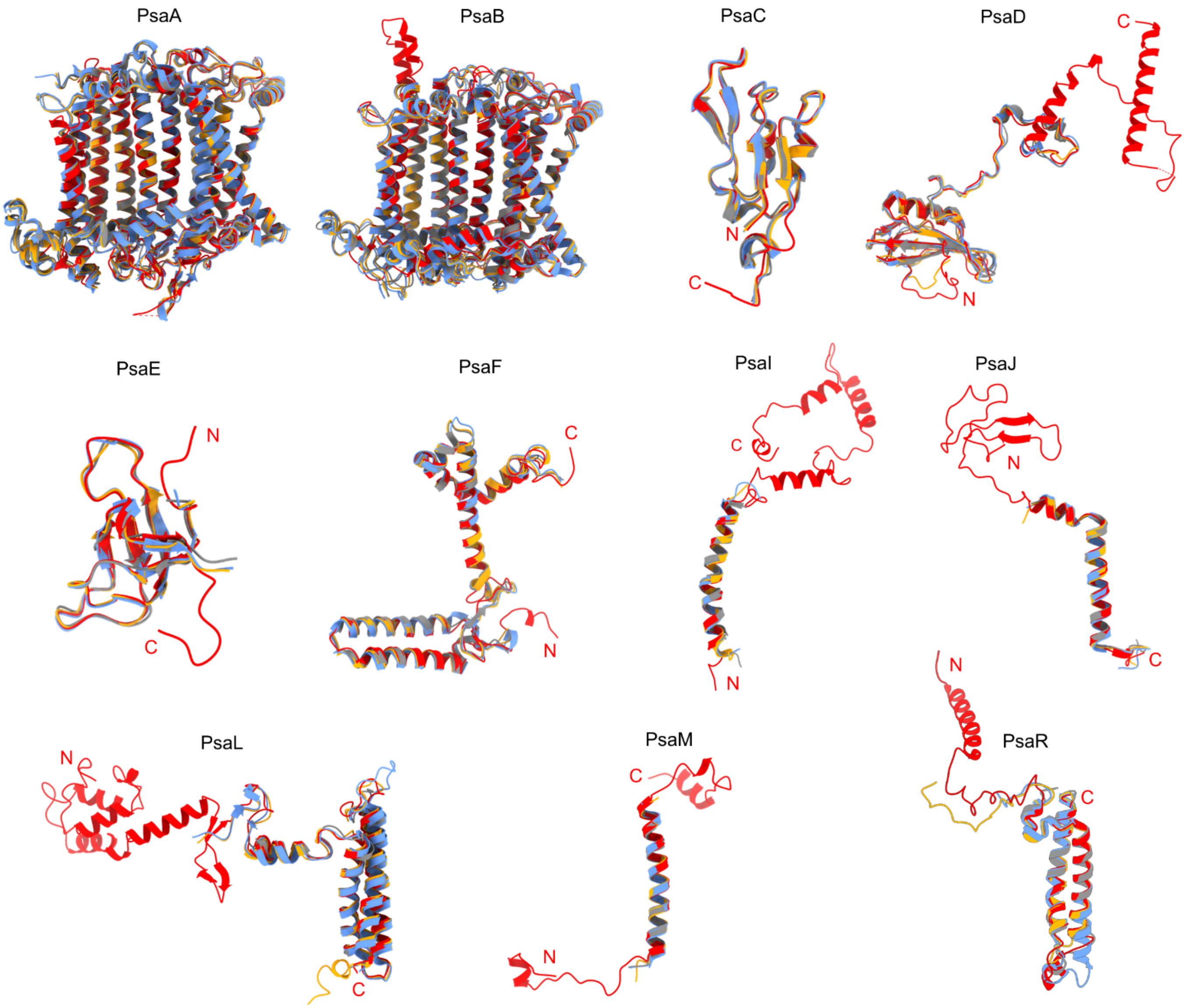

Supplementary Fig. 4. Structural comparison of the PSI core subunits from *Symbiodinium* (red), red algae (gray, PDB: 7Y5E), cryptophyte (orange, PDB: 7Y7B), and diatom (blue, PDB: 6LY5).

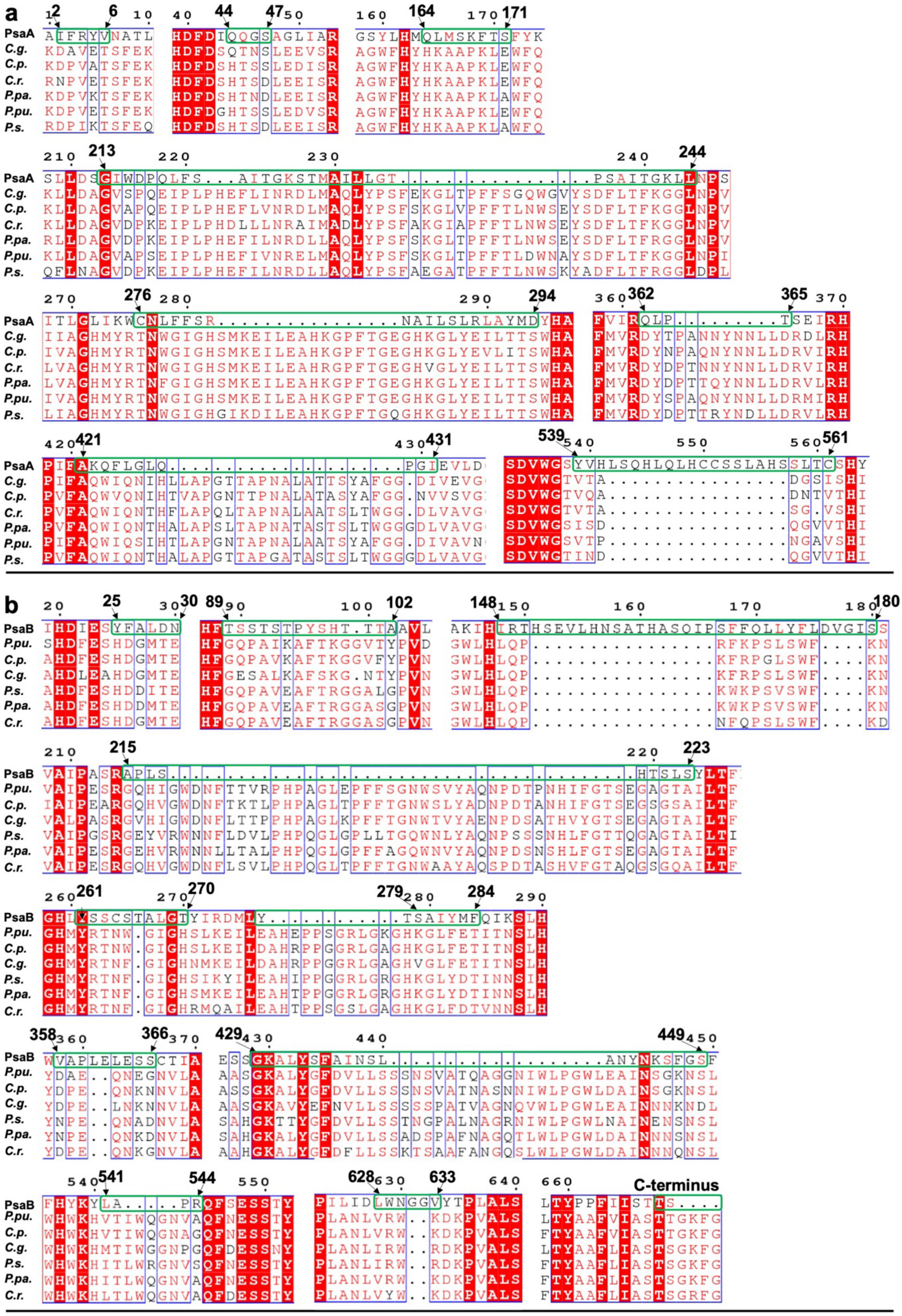
a
PsaA
C.g.
C.p.
C.r.
P.pa.
P.pu.
P.s.
b
PsaB
C-terminus

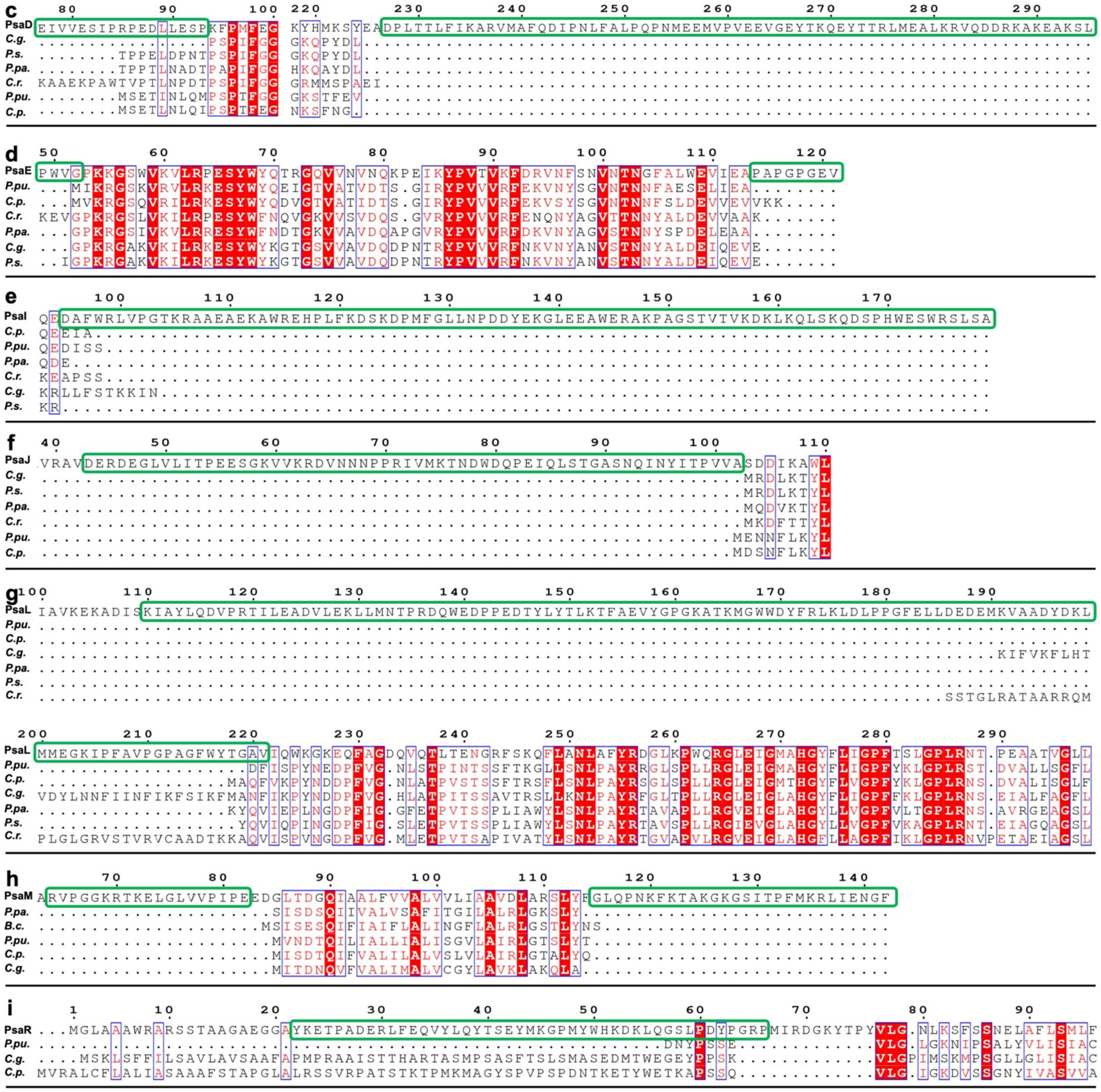

Supplementary Fig. 5. Sequence alignments of the PSI–AcpPCI core subunits and their homologous sequences from red algae, cryptophyte, diatom, green algae, moss, and land plants. a-i, Sequence comparison of the *Symbiodinium* PsaA (**a**), PsaB (**b**), PsaD (**c**), PsaE (**d**), PsaI (**e**), PsaJ (**f**), PsaL (**g**), PsaM (**h**), and PsaR (**i**) with their homologous sequences respectively. The loops of PsaA and PsaB that have conformational changes are indicated by arrows. The extended terminal domains are highlighted with green boxes. Fully conserved residues are shaded in red, and similar amino acids are highlighted by blue frames. *Porphyridium purpureum*: *P.pu.*, *Chroomonas placoidea*, *C.p.*, *Chaetoceros gracilis*: *C.g.*, *Chlamydomonas reinhardtii*: *C.r.*, *Bryopsis corticulans*: *B.c.*, *Physcomitrium patens*: *P.pa.*, *Pisum sativum*: *P.s.*.

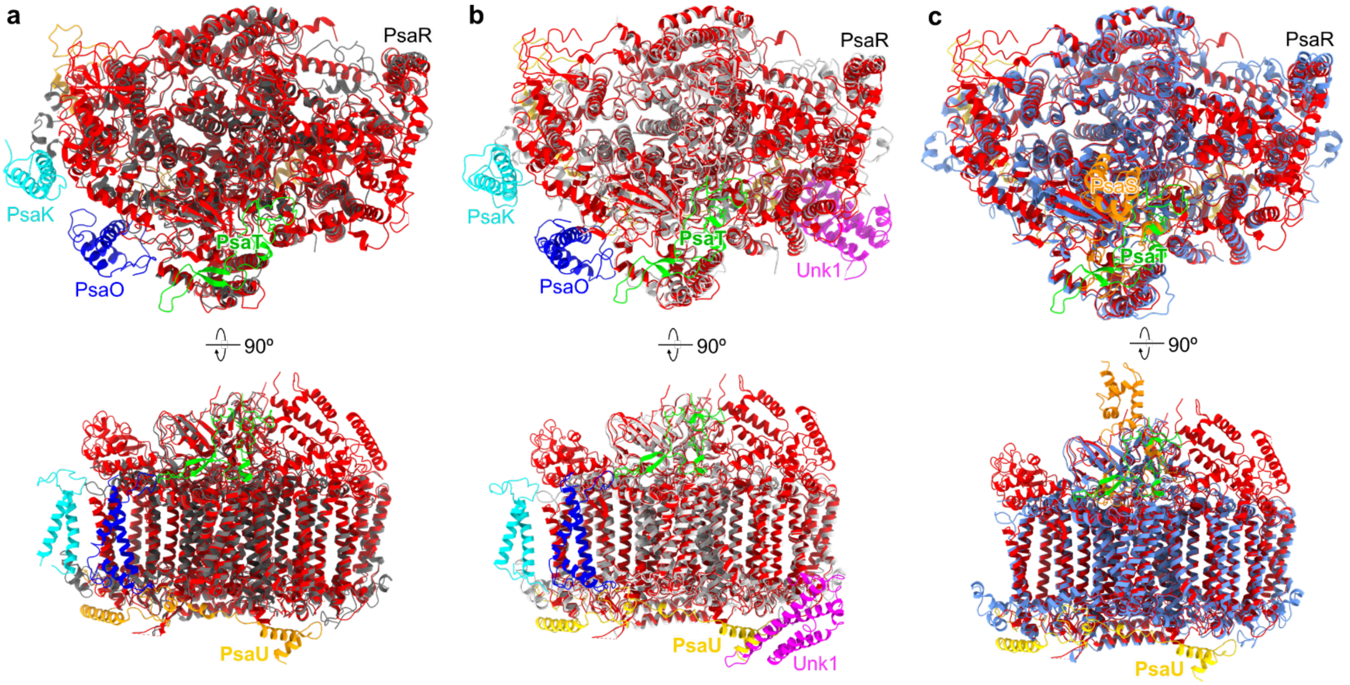

Supplementary Fig. 6. Comparison of the PSI cores of *Symbiodinium*, cryptophyte, red algae, and diatom. a-c, Superposition of the *Symbiodinium* PSI core (red) with red algal PSI core (dim gray, PDB: 7Y5E) (**a**), cryptophytic PSI core (light gray, PDB: 7Y7B) (**b**), and diatom PSI core (blue, PDB: 6LY5) (**c**). PsaT and PsaU, which are absent in other PSI core, are labeled. PsaK, PsaO, Unk1 and PsaS, which are absent in *Symbiodinium* PSI core, are indicated. PsaR which exists in all the four PSI core is labeled.

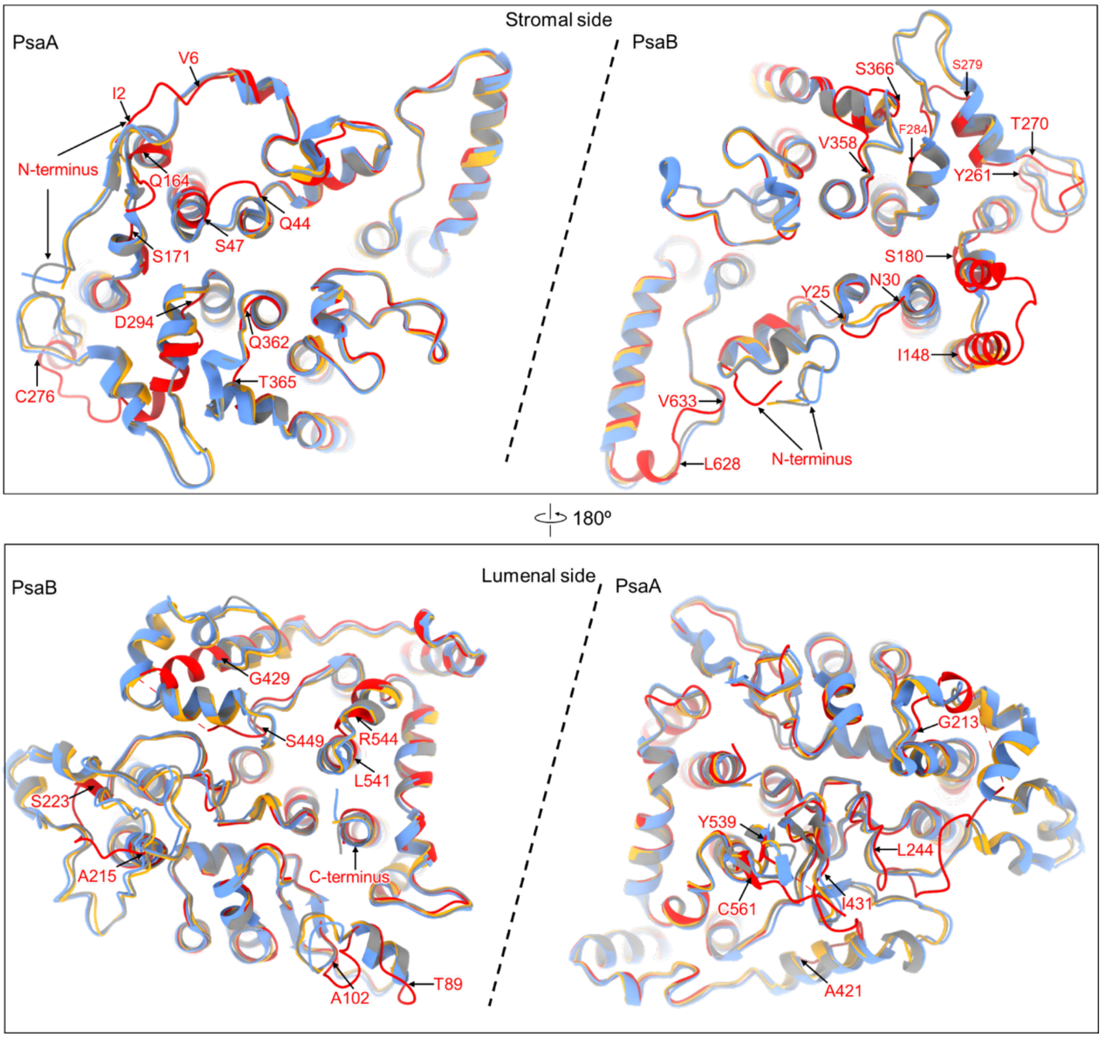

Supplementary Fig. 7. Structural comparison of the PsaA and PsaB subunits from *Symbiodinium* (red), red algae (gray, PDB: 7Y5E), cryptophyte (orange, PDB: 7Y7B), and diatom (blue, PDB: 6LY5) viewed from the stromal side (up panel) and lumenal side (bottom panel). The structural changes of the loops between transmembrane helices of *Symbiodinium* PsaA and PsaB are indicated by arrows.

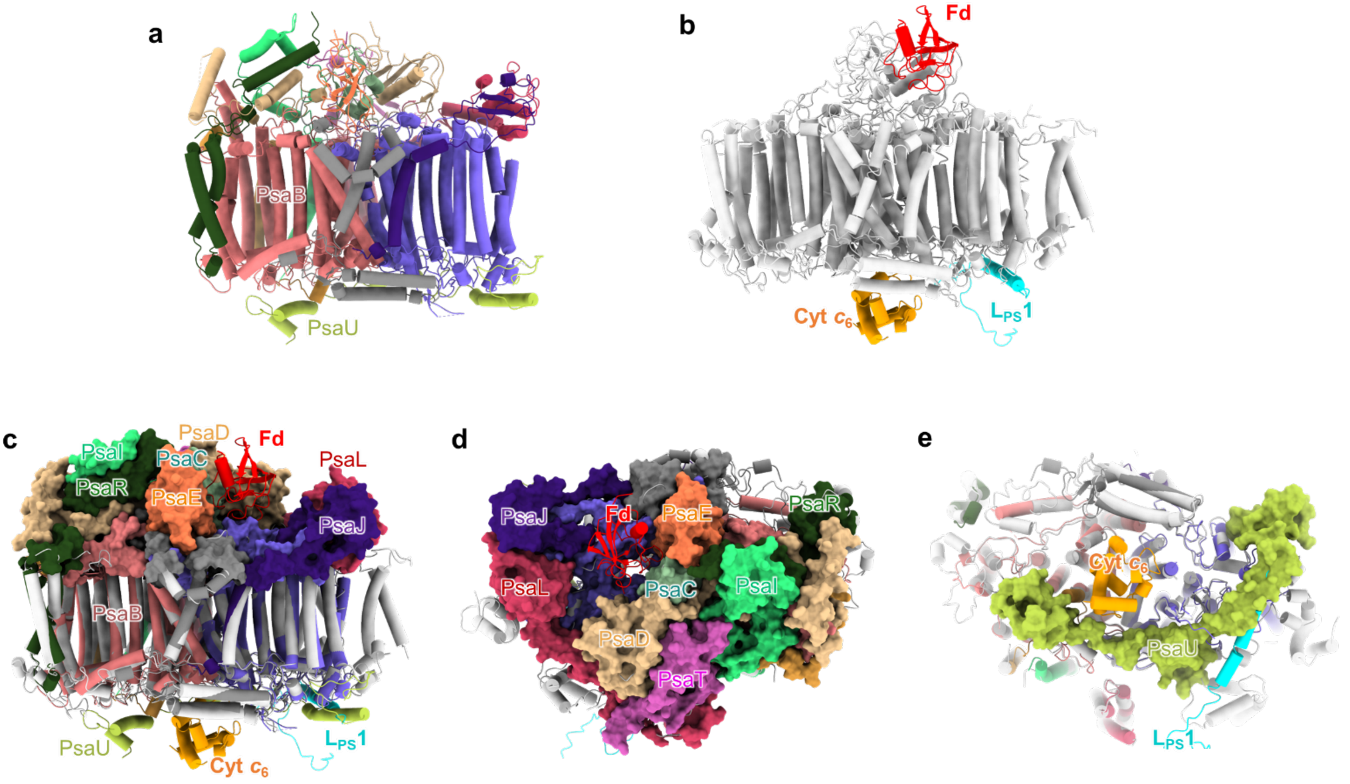

Supplementary Fig. 8. The surface structures of *Symbiodinium* PSI core and the supposed binding positions of ferredoxin (Fd) and cytochrome c_6 (Cyt c_6). **a**, Side view of the *Symbiodinium* PSI core. **b**, Side view of the red algal PSI core with Fd and Cyt c_6. PDB: 7Y5E. **c-e**, Superposition of the *Symbiodinium* PSI core (**a**) with red algal PSI core (**b**) viewed from side (**c**), top (**d**) and bottom (**e**), showing the possible binding positions of Fd and Cyt c_6 in *Symbiodinium* PSI core. The structures at stromal side and of PsaU in **e** are shown as surface.

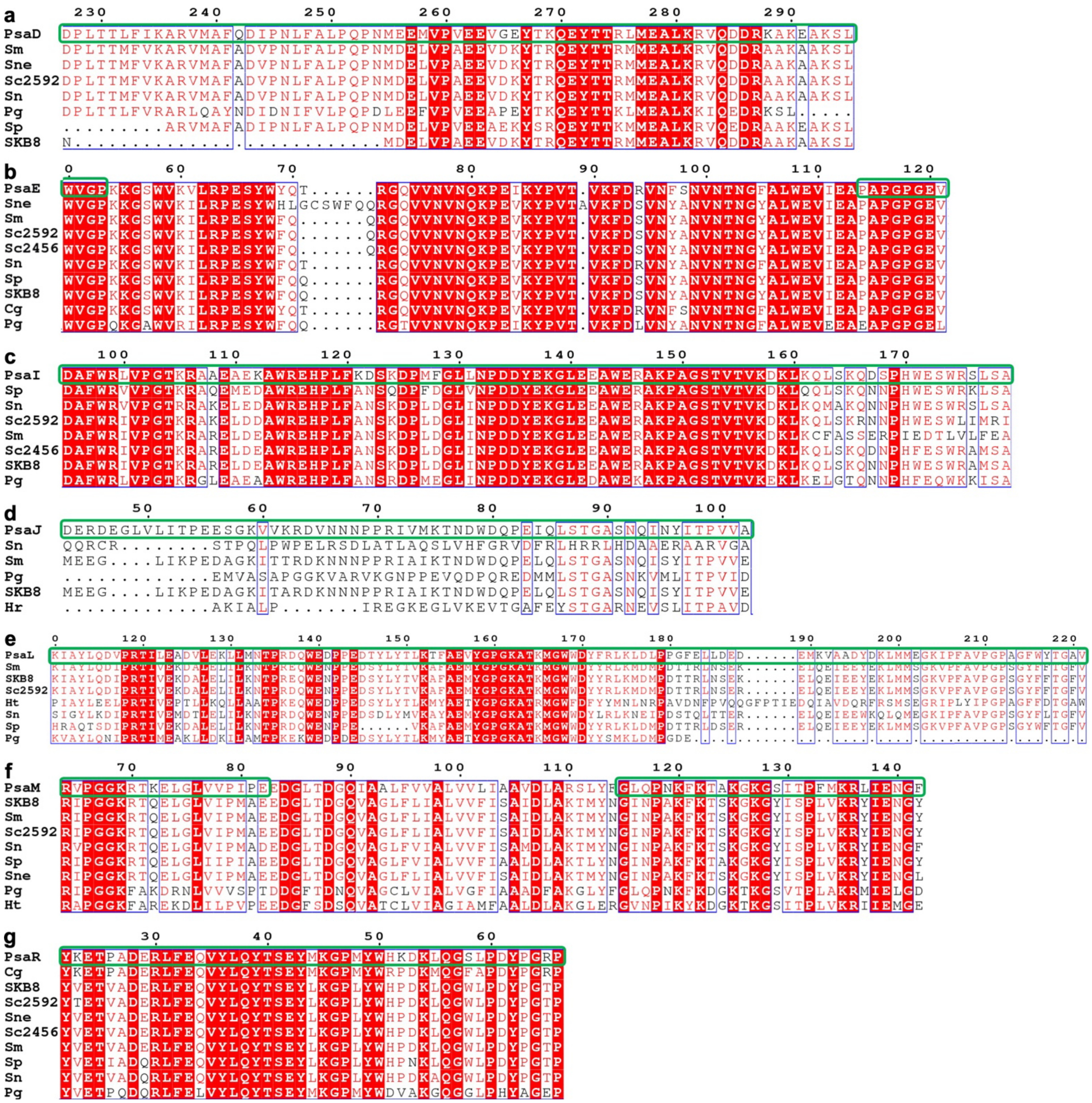
a
PsaD
Sm
Sne
Sc2592
Sn
Pg
Sp
SKB8
b
PsaE
Sne
Sm
Sc2592
Sc2456
Sn
Sp
SKB8
Cg
Pg
c
PsaI
Sp
Sn
Sc2592
Sm
Sc2456
SKB8
Pg
d
PsaJ
Sn
Sm
Pg
SKB8
Hr
e
PsaL
Sm
SKB8
Sc2592
Ht
Sn
Sp
Pg
f
PsaM
SKB8
Sm
Sc2592
Sn
Sp
Sne
Pg
Ht
g
PsaR
Cg
SKB8
Sc2592
Sne
Sc2456
Sm
Sp
Sn
Pg

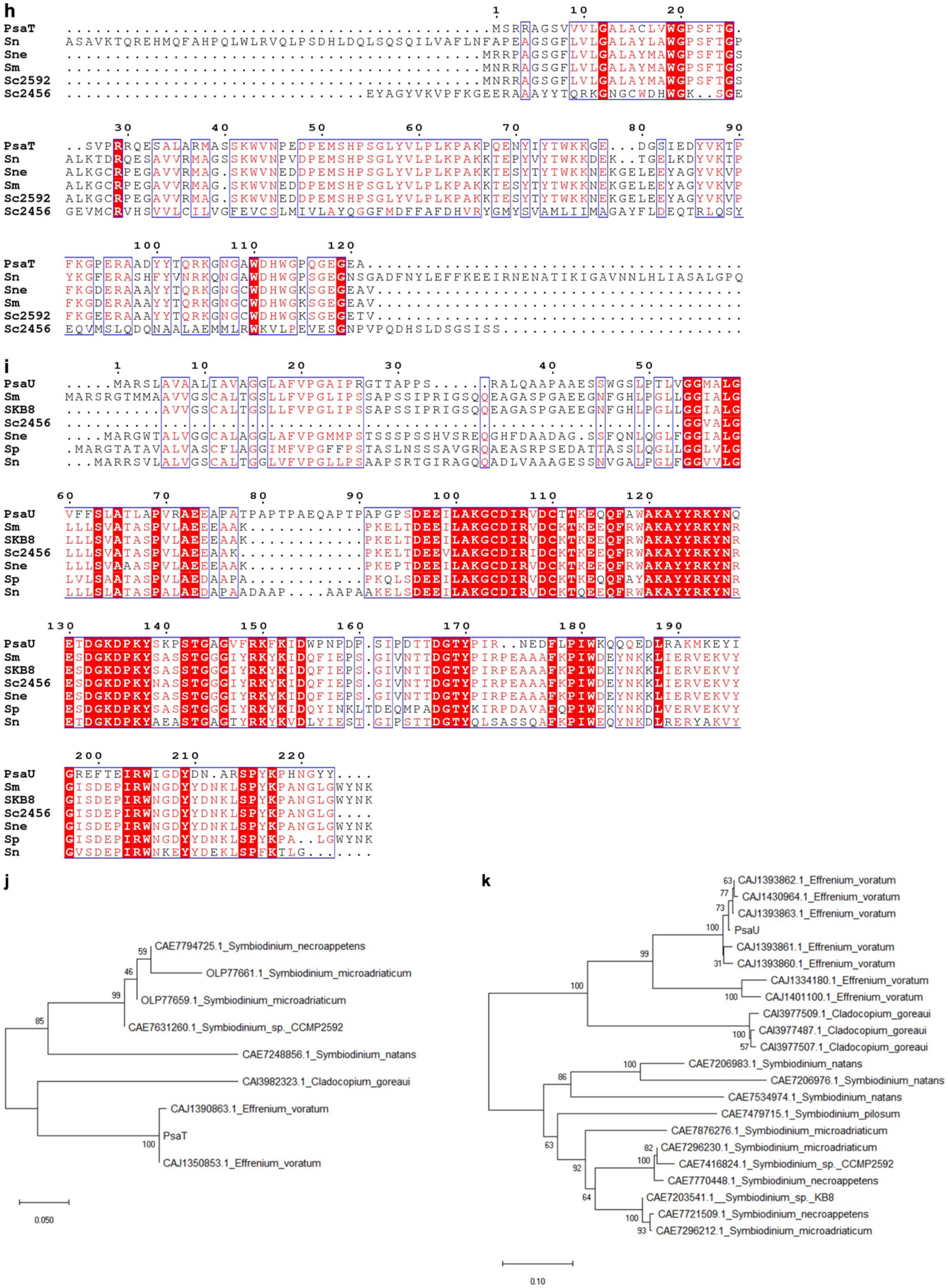

h
PsaT
Sn
Sne
Sm
Sc2592
Sc2456
i
PsaU
Sm
SKB8
Sc2456
Sne
Sp
Sn
j
CAE7794725.1_Symbiodinium_necroappetens
OLP77661.1_Symbiodinium_microadriaticum
OLP77659.1_Symbiodinium_microadriaticum
CAE7631260.1_Symbiodinium_sp._CCMP2592
CAE7248856.1_Symbiodinium_natans
CAI3982323.1_Cladocopium_goreaui
CAJ1390863.1_Effrenium_voratum
PsaT
CAJ1350853.1_Effrenium_voratum
0.050
k
CAJ1393862.1_Effrenium_voratum
CAJ1430964.1_Effrenium_voratum
CAJ1393863.1_Effrenium_voratum
PsaU
CAJ1393861.1_Effrenium_voratum
CAJ1393860.1_Effrenium_voratum
CAJ1334180.1_Effrenium_voratum
CAJ1401100.1_Effrenium_voratum
CAI3977509.1_Cladocopium_goreaui
CAI3977487.1_Cladocopium_goreaui
CAI3977507.1_Cladocopium_goreaui
CAE7206983.1_Symbiodinium_natans
CAE7206976.1_Symbiodinium_natans
CAE7534974.1_Symbiodinium_natans
CAE7479715.1_Symbiodinium_pilosum
CAE7876276.1_Symbiodinium_microadriaticum
CAE7296230.1_Symbiodinium_microadriaticum
CAE7416824.1_Symbiodinium_sp._CCMP2592
CAE7770448.1_Symbiodinium_necroappetens
CAE7203541.1__Symbiodinium_sp._KB8
CAE7721509.1_Symbiodinium_necroappetens
CAE7296212.1_Symbiodinium_microadriaticum
0.10

Supplementary Fig. 9. Sequence alignments of the specific core subunits with additional terminal domains, the PsaT and PsaU subunits from PSI–AcpPCI and their homologous sequences from other algae. a-i, Sequence comparison of PsaD (**a**), PsaE (**b**), PsaI (**c**), PsaJ (**d**), PsaL (**e**), PsaM (**f**), PsaR (**g**), PsaT (**h**), and PsaU (**i**) with their homologous sequences respectively. The additional terminal domains are highlighted with green boxes. Fully conserved residues are shaded in red, and similar amino acids are highlighted by blue frames. *Symbiodinium microadriaticum*: Sm, *Symbiodinium natans*: Sn, *Symbiodinium necroappetens*: Sne, *Symbiodinium pilosum*: Sp, *Symbiodinium sp.* CCMP2592: Sc2592, *Symbiodinium sp.* CCMP2456: Sc2456, *Symbiodinium sp.* KB8: SKB8, *Cladocopium goreaui*: Cg, *Polarella glacialis*: Pg, *Heterocapsa rotundata*: Hr, *Heterocapsa triquetra*: Ht. **j**, Phylogenetic tree of PsaT and its homologous sequences. **k**, Phylogenetic tree of PsaU and its homologous sequences. The neighbor-joining tree was based on amino acid sequences of LHCIs. The phylogenetic trees were built with the Poisson model, and a bootstrap test (1,000 replicates) was conducted.

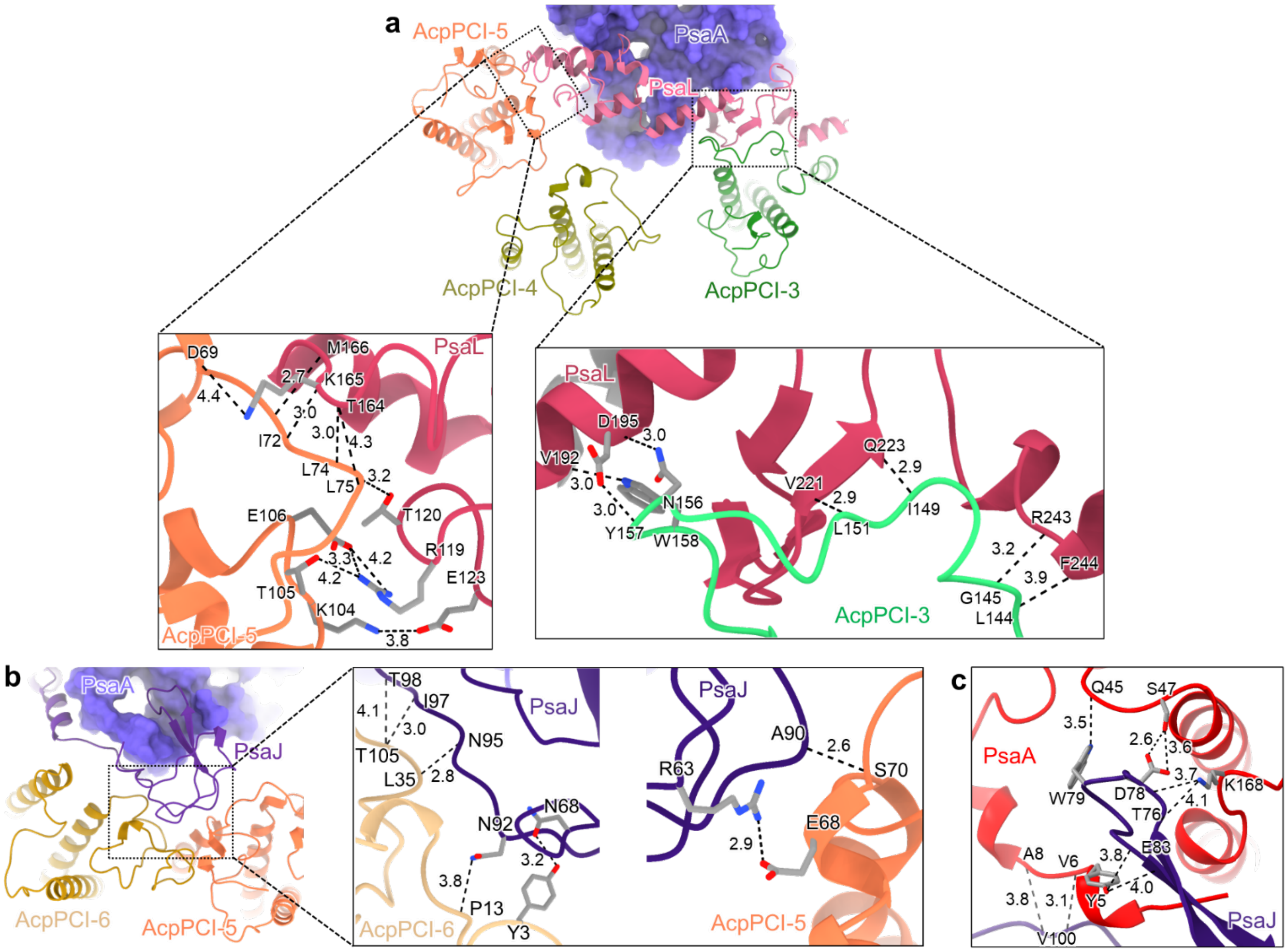

Supplementary Fig. 10. Interactions between the extended terminal loops of PsaL, PsaJ and surrounding subunits. a, Interactions between the extended N-terminal loop of PsaL and adjacent AcpPCIs. Squared areas are enlarged, showing the detailed hydrogen bond interactions. **b**, Interactions between the extended N-terminal loop of PsaJ and adjacent AcpPCIs. Squared areas are enlarged, showing the detailed hydrogen bond interactions. **c**, Detailed information of interactions between PsaJ and the changed loop structures of PsaA indicated in Fig 2a. Interactions are indicated by black dashed lines with distances labeled in Å.

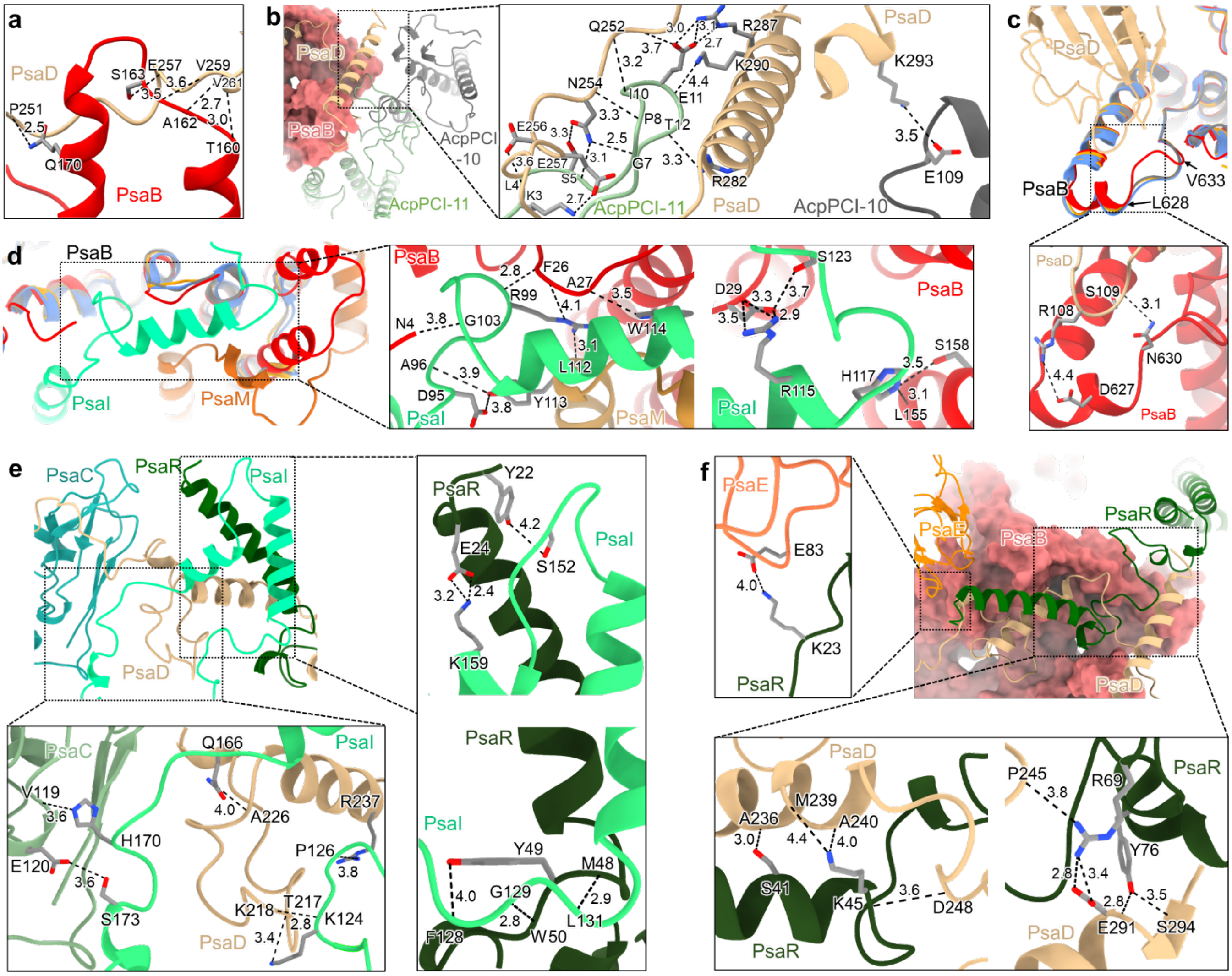

Supplementary Fig. 11. Interactions between the extended terminal loops of PsaD, PsaI, PsaR and surrounding subunits. a, Detailed information of interactions between the extended C-terminal loops PsaD and the changed loop structures of PsaB indicated in Fig. 2b. **b**, Interactions between the extended C-terminal loops of PsaD and adjacent AcpPCIs. Squared areas are enlarged, showing the detailed hydrogen bond interactions. **c**, Detailed information of interactions between the extended N-terminal loops PsaD and the changed loop structures of PsaB indicated in Fig. 2b. The PsaB subunits of *Symbiodinium*, red algae, cryptophyte, and diatom are colored red, gray, orange, and blue, respectively. **d-e**, Interactions between the extended C-terminal loops of PsaI and adjacent subunits. Squared areas are enlarged, showing the detailed hydrogen bond interactions. **f**, Interactions between the extended N-terminal loops of PsaR and adjacent subunits. Squared areas are enlarged, showing the detailed hydrogen bond interactions. Interactions are indicated by black dashed lines with distances labeled in Å.

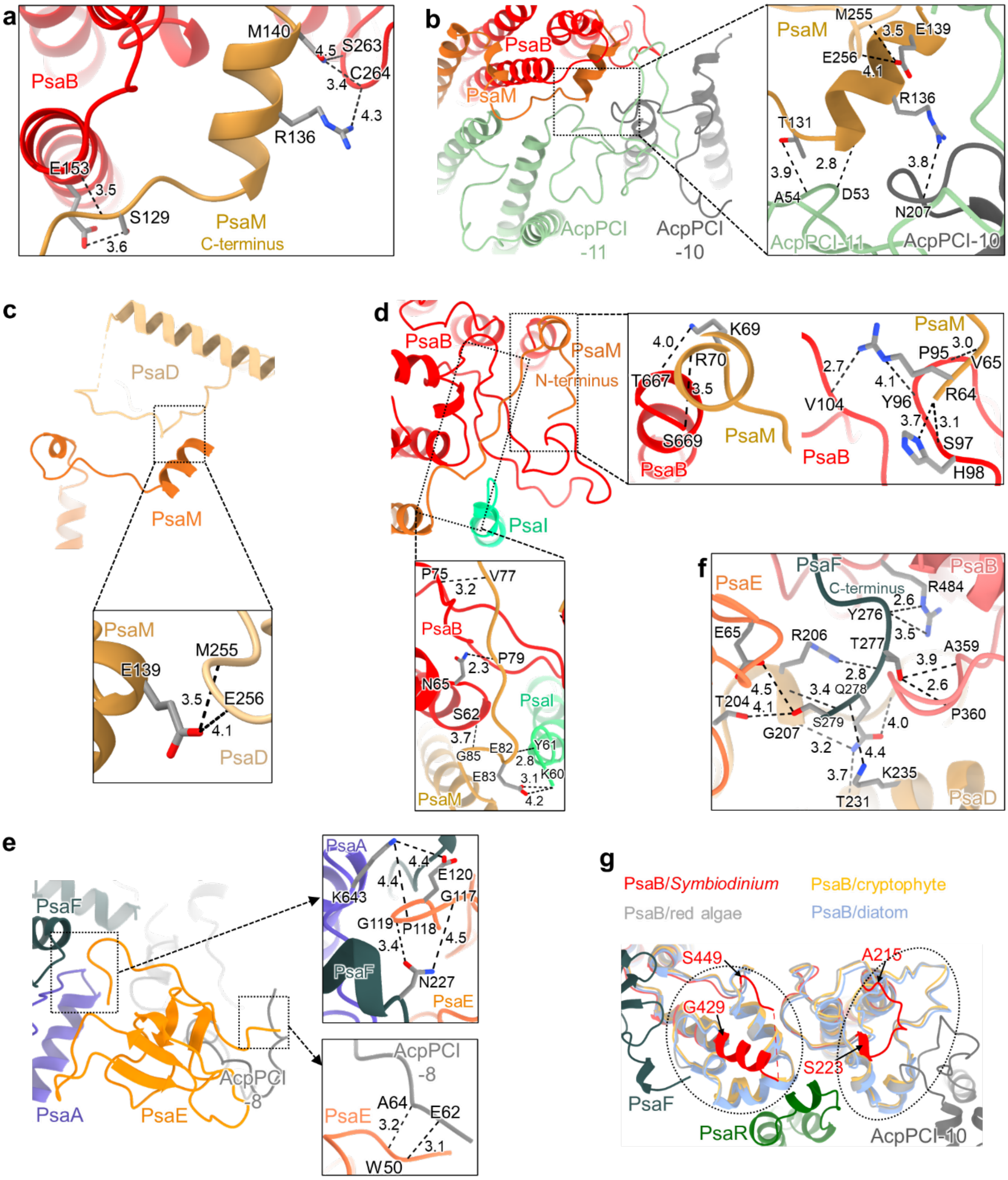

Supplementary Fig. 12. Interactions between the extended terminal loops of PsaM, PsaE, PsaF and surrounding subunits. a, Detailed information of interactions between extended C-terminal loop of PsaM and the changed loop structures of PsaB indicated in Fig. 2**d**. **b-c**, Interactions between the extended C-terminal loop of PsaM and adjacent AcpPCIs (**b**) and extended N-terminal loop of PsaD (**c**). Squared areas are enlarged, showing the detailed hydrogen bond interactions. **d**, Interactions between the extended N-terminal loop of PsaM and adjacent subunits. Squared areas are enlarged, showing the detailed hydrogen bond interactions. **e-f**, Interactions between the extended terminal loops of PsaE, PsaF and adjacent subunits. Squared areas are enlarged, showing the detailed hydrogen bond interactions. **g**, The reductions of the loops of PsaB (A215-S223 and G429-S449) indicated with dashed ovals, avoid clashes with the extended N-terminus of PsaF and lumenal loops of PsaR and AcpPCI-10. The PsaB subunits of *Symbiodinium*, red algae, cryptophyte, and diatom are colored red, gray, orange and blue, respectively. Interactions are indicated by black dashed lines with distances labeled in Å.

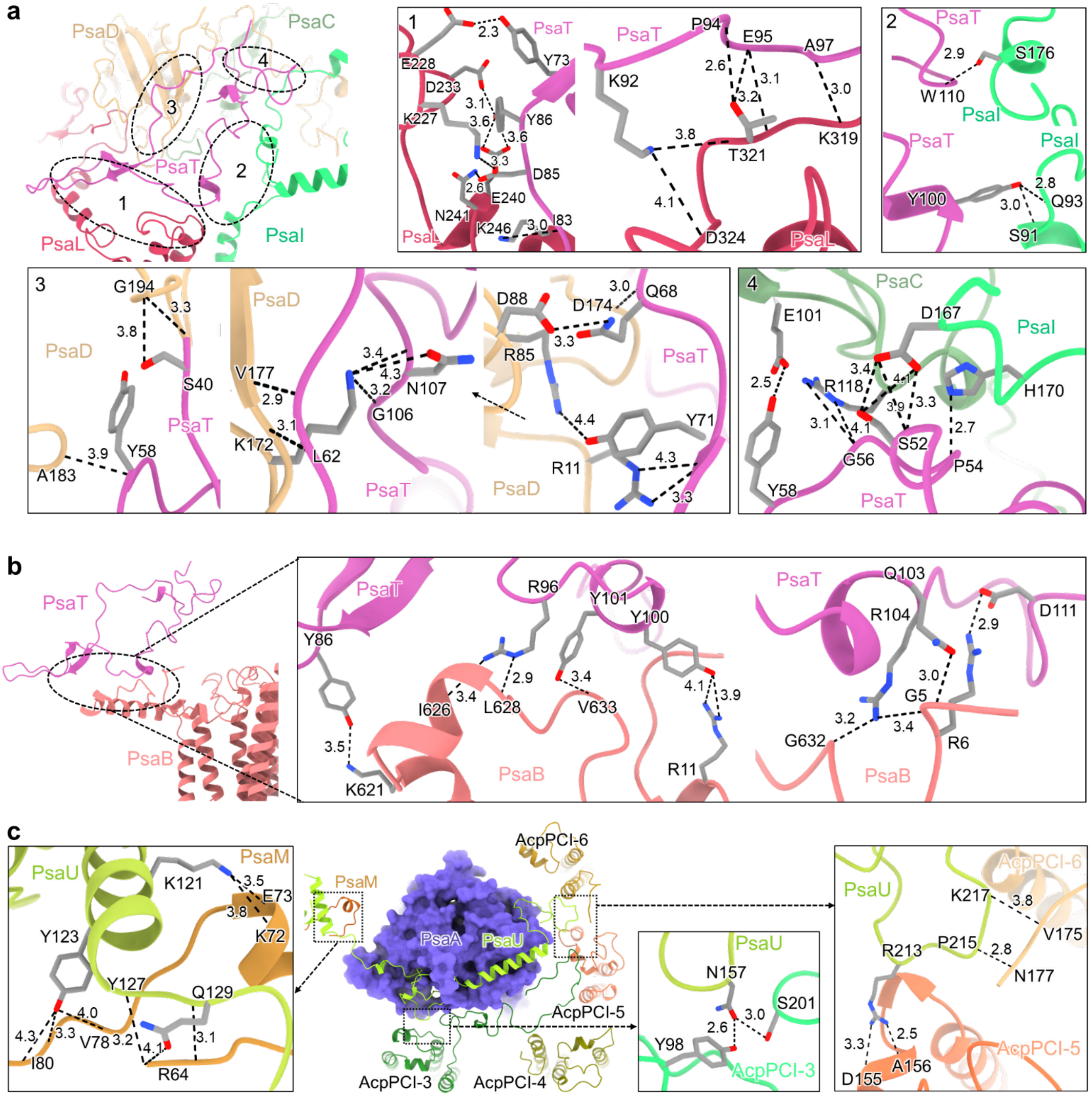

Supplementary Fig. 13. Locations and structures of subunits PsaT, PsaU and their interactions with surrounding subunits. a-b, Interactions between PsaT and adjacent subunits. Circled areas are enlarged, showing the detailed hydrogen bond interactions. **c**, Interactions between PsaU and adjacent AcpPCIs and the extended N-terminal loop of PsaM. Squared areas are enlarged, showing the detailed hydrogen bond interactions. Interactions are indicated by black dashed lines with distances labeled in Å.

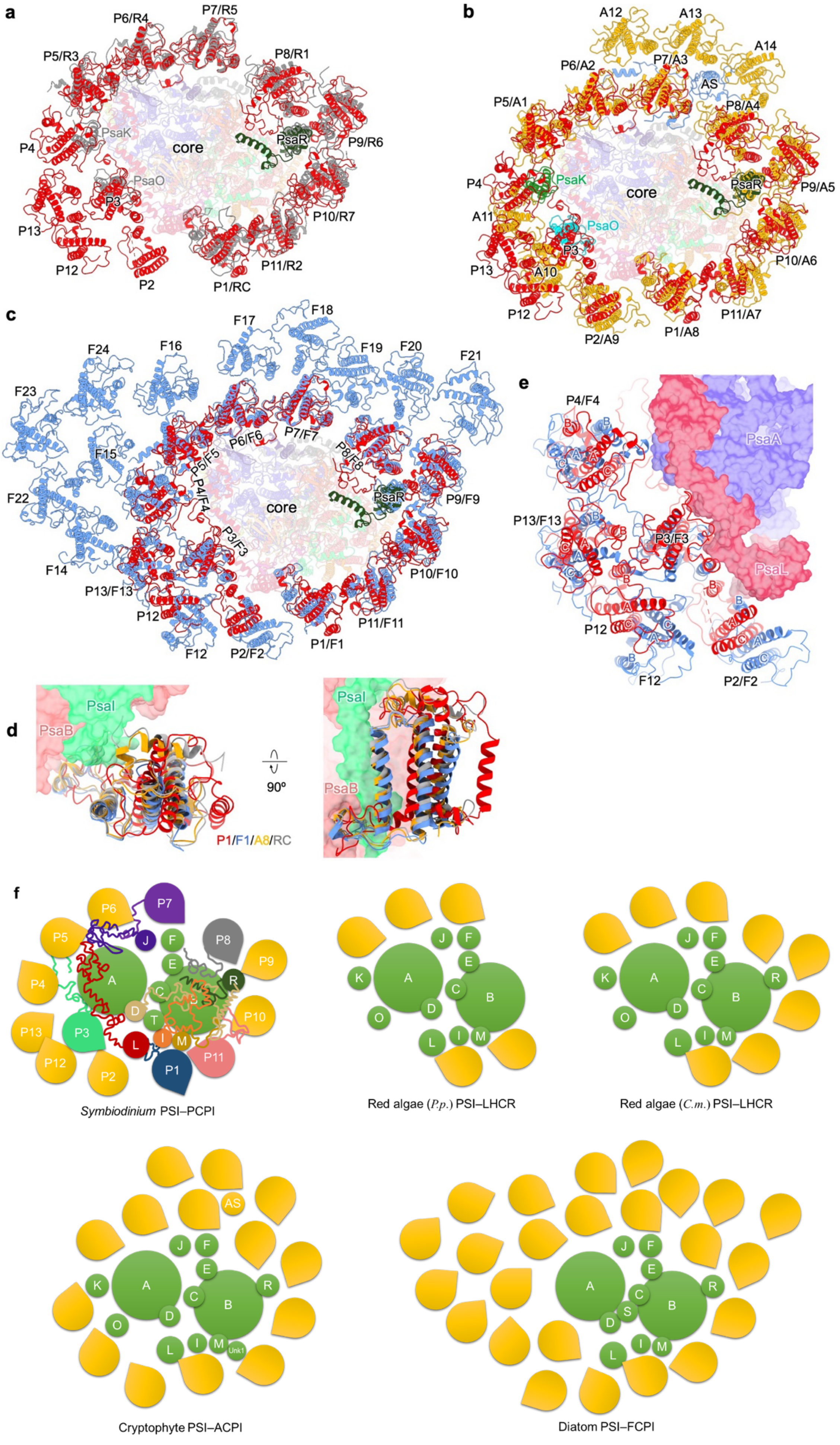
a
P6/R4
P7/R5
P5/R3
P8/R1
PsaK
core
PsaR
P9/R6
P4
PsaO
P3
P10/R7
P13
P12
P2
P1/RC
P11/R2
b
A12
A13
A14
P6/A2
P7/A3
AS
P5/A1
P8/A4
P4
PsaK
core
PsaR
P9/A5
A11
PsaO
P3
P13
A10
P10/A6
P12
P2/A9
P1/A8
P11/A7
c
F17
F18
F24
F16
F19
F20
F21
F23
F15
P5/F5
P6/F6
P7/F7
P8/F8
P4/F4
PsaR
F22
core
P9/F9
P3/F3
F14
P13/F13
P10/F10
P12
P11/F11
F12
P2/F2
P1/F1
e
P4/F4
PsaA
P13/F13
P3/F3
PsaL
P12
F12
P2/F2
d
PsaI
PsaB
90º
P1/F1/A8/RC
PsaI
PsaB
f
P7
P6
P5
J
F
P8
P9
P4
A
E
R
C
D
T
P10
P13
P3
I
M
P12
L
P11
P2
P1
Symbiodinium PSI–PCPI
J
F
K
A
E
C
B
O
D
L
I
M
Red algae (*P.p.*) PSI–LHCR
J
F
K
A
E
R
C
B
O
D
L
I
M
Red algae (*C.m.*) PSI–LHCR
AS
J
F
E
K
A
R
C
B
O
D
L
I
M
Unk1
Cryptophyte PSI–ACPI
J
F
E
A
R
C
S
B
D
L
I
M
Diatom PSI–FCPI

Supplementary Fig. 14. Comparison of the arrangements of AcpPCIs in *Symbiodinium* PSI–AcpPCI with those in red algae PSI–LHCR (PDB: *Porphyridium purpureum P.p.*, 7Y5E; *Cyanidioschyzon merolae C.m.*, 5ZGB), cryptophyte PSI–ACPI (PDB: 7Y7B), and diatom PSI–FCPI (PDB: 6LY5). **a**, Superposition of the PSI–AcpPCI structure (red) with red algal PSI–LHCR structure (gray). PsaK and PsaO of red algae and PsaR are labeled. Letters of P1-P13 and R1-R7 indicate AcpPCI1-14 and LHCR1-7, respectively. RC indicates RedCAP. **b**, Superposition of the PSI–AcpPCI structure (red) with cryptophyte PSI–ACPI structure (orange). PsaK and PsaO of cryptophyte and PsaR are labeled. Letters of A1-A14 indicate ACPI1-14, respectively. **c**, Superposition of the PSI–AcpPCI structure (red) with diatom PSI–FCPI structure (blue). PsaR are labeled. Letters of F1-F24 indicate FCPI1-24, respectively. **d**, Rotation of the AcpPCI-1 (red) compared with RedCAP (gray), ACPI-1 (orange) and FCPI-1 (blue). **e**, Shift of AcpPCI-2/3/4/12/13 (red) compared with FCPI-2/3/4/12/13 (blue) viewed from the stromal side. The transmembrane helices of AcpPCIs and FCPIs are labeled. **f,** Schematic models of the organizations of the PSI core (green) and LHC (orange) components in *Symbiodinium* PSI–AcpPCI, red algae PSI–LHCR, cryptophyte PSI–ACPI, and diatom PSI–FCPI. Letters of A-T and AS indicate PsaA-PsaT and ACPI-S, respectively. The extended termini of the PsaD/I/J/L/M/R and AcpPCI-1/3/7/8/11 are highlighted.

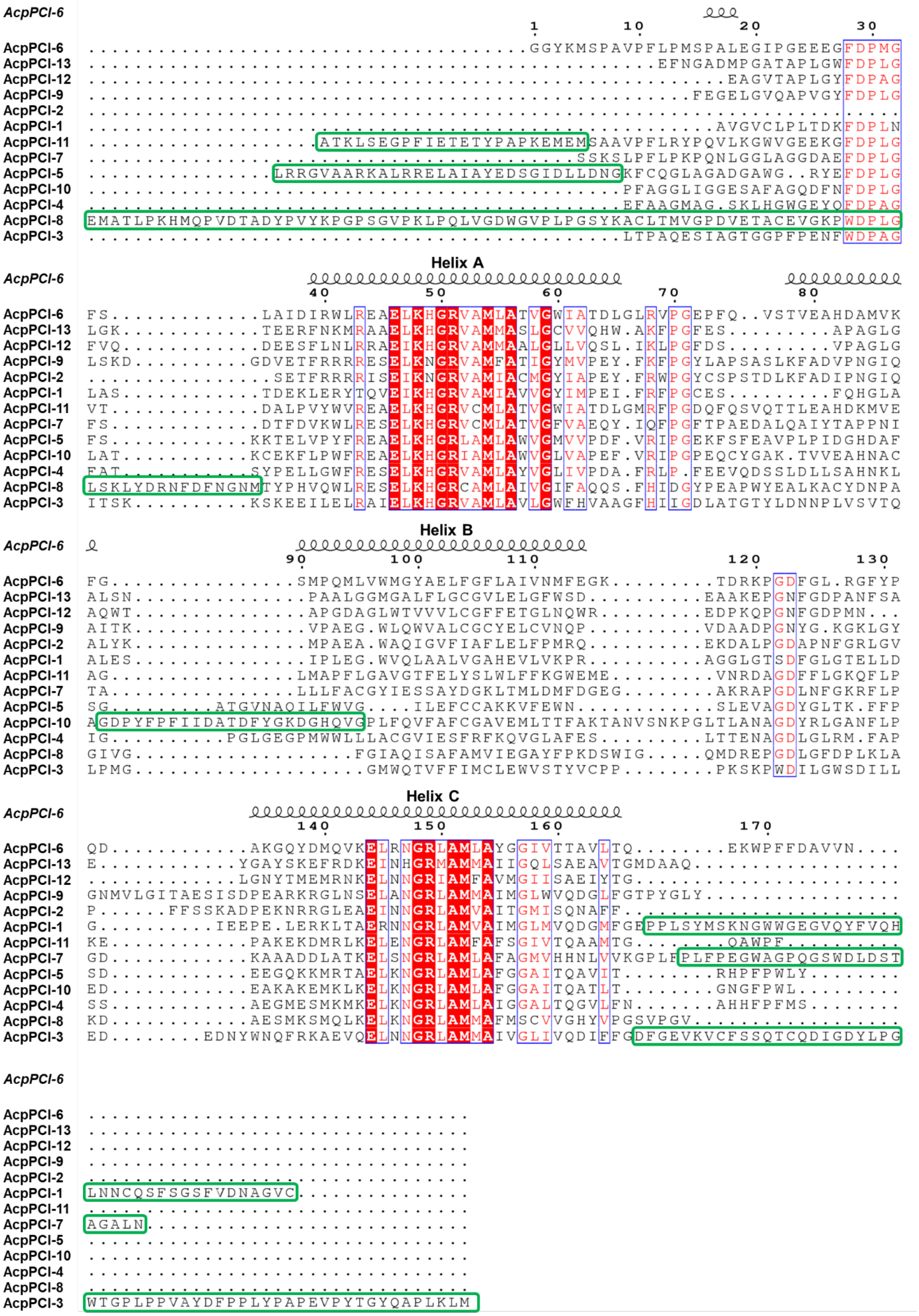

Supplementary Fig. 15. Sequence alignment of AcpPCIs from *Symbiodinium* PSI–AcpPCI. The secondary structure is shown above the sequences. Fully conserved residues are shaded in red, and similar amino acids are highlighted by blue frames.

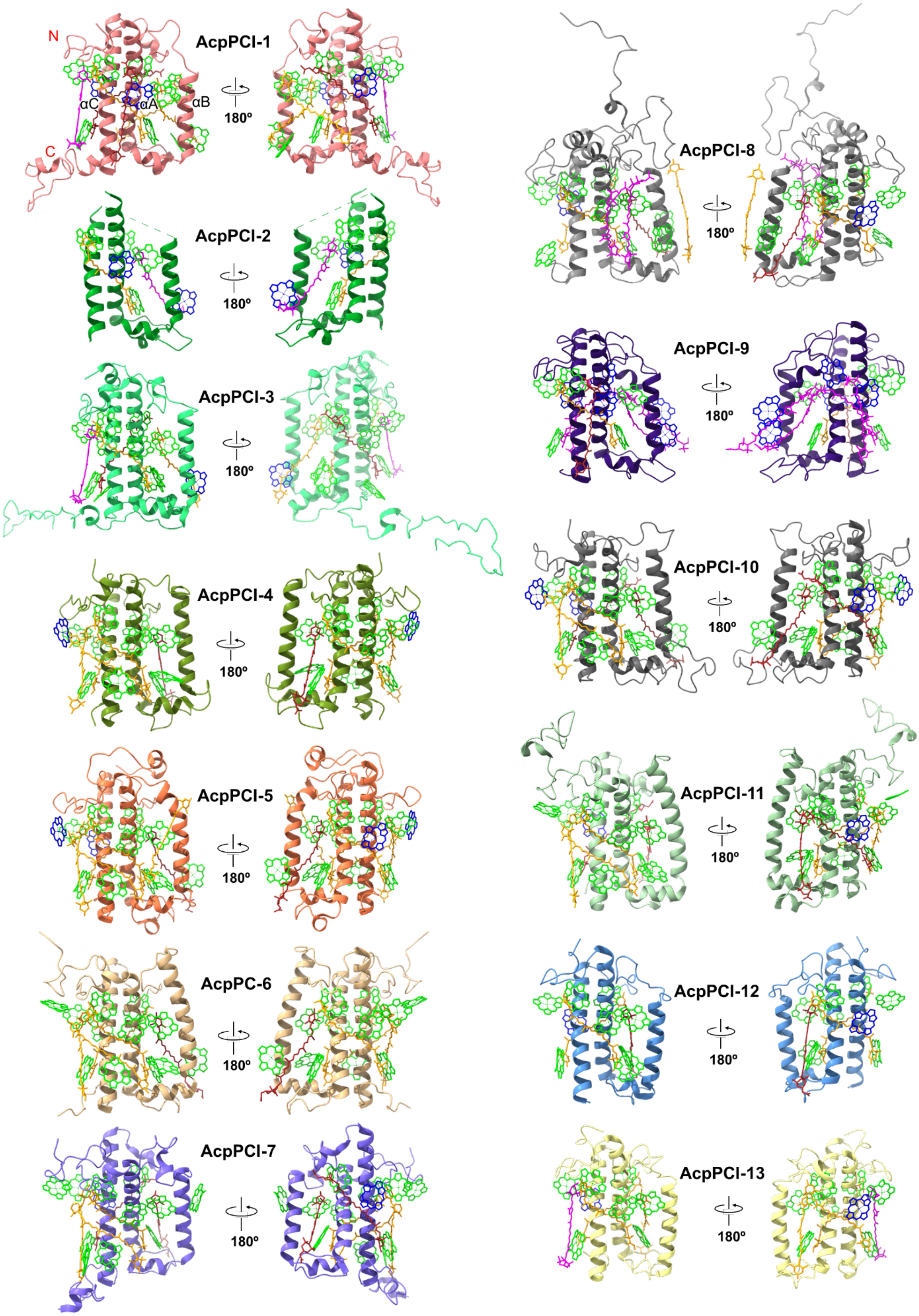

Supplementary Fig. 16. Structures of individual AcpPCI subunits. Chl *a*, Chl *c*, Diadinoxanthin, Peridinin, and Dinoxanthin are colored in green, blue, orange, magenta, and brown, respectively. The phytol chains of Chls are omitted.

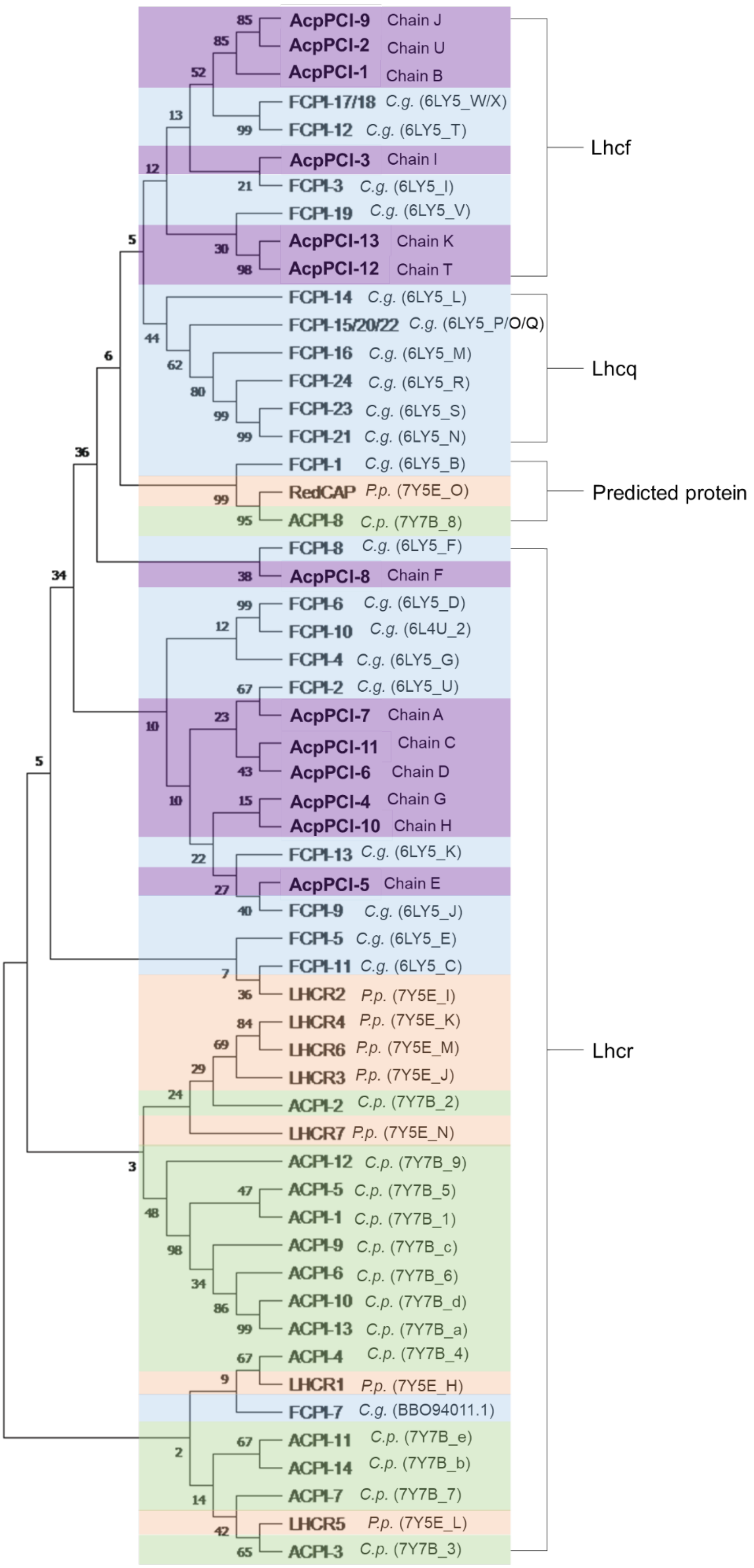

Supplementary Fig. 17. Phylogenetic tree of LHCIs in *Symbiodinium* (purple), cryptophytic alga *C. placoidea* (*C.p.*) (green), red alga *P. purpureum* (*P.p.*) (orange), and diatom *C. gracilis* (*C.g.*) (blue). The neighbor-joining tree was based on amino acid sequences of LHCIs. The tree was built with the Poisson model using 485 amino acid residues, and a bootstrap test (1,000 replicates) was conducted.

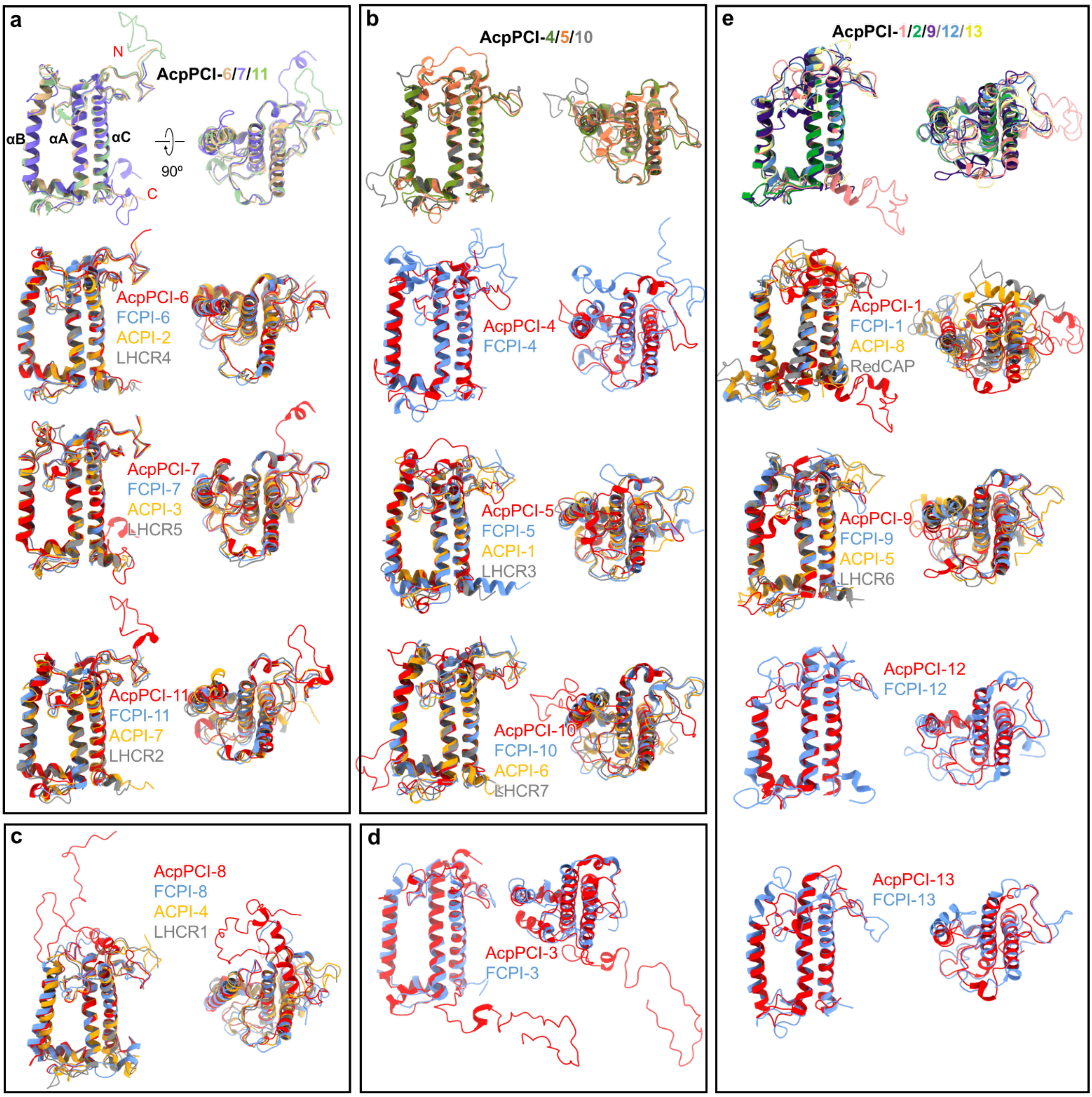

Supplementary Fig. 18. Structural comparison of the AcpPCIs and between AcpPCIs and LHCRs from red algae (PDB: 7Y5E), ACPIs from cryptophyte (PDB: 7Y7B) and FCPIs from diatom (PDB: 6LY5). a, Superposition of AcpPCI-6/7/11 and comparisons with the corresponding LHCRs, ACPIs and FCPIs. **b**, Superposition of AcpPCI-4/5/10 and comparisons with the corresponding LHCRs, ACPIs and FCPIs. **c**, Superposition of AcpPCI-8 and the corresponding FCPI-8, ACPI-4 and LHCR1. **d**, Superposition of AcpPCI-3 and the corresponding FCPI-3. **e**, Superposition of AcpPCI-1/2/9/12/13 and comparisons with the corresponding LHCRs, ACPIs and FCPIs.

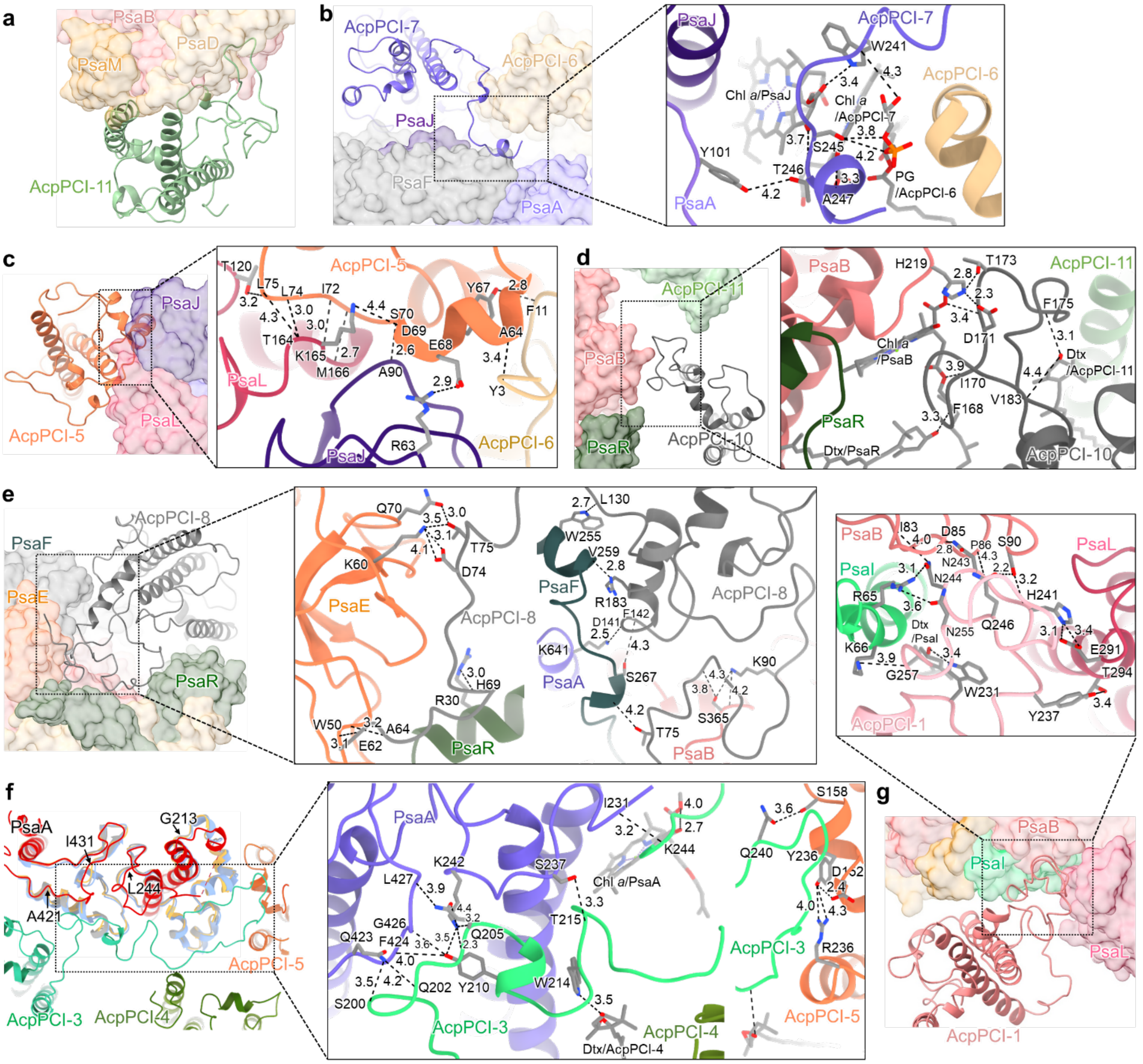

Supplementary Fig. 19. Interactions between the extended terminal loops of AcpPCIs and surrounding subunits. a, The interactions between the extended N-terminal loop of AcpPCI-11 and extended C-terminal loops of PsaD. **b**, The interactions of the extended C-terminal loop of AcpPCI-7 with PsaA, PsaJ and AcpPCI-6. **c**, The interactions of the extended N-terminal loop of AcpPCI-5 with PsaL, PsaJ and AcpPCI-6. **d**, The interactions of the extended C-terminal loop of AcpPCI-10 with PsaB, PsaR and AcpPCI-11. **e**, The interactions of the extended N-terminal loop of AcpPCI-8 with PsaA, PsaB, PsaE, PsaF and PsaR. **f**, The interactions of the extended C-terminal loop of AcpPCI-3 with PsaA and AcpPCI-3/4/5. **g**, The interactions of the extended C-terminal loop of AcpPCI-1 with PsaB, PsaL and PsaI. Interactions are indicated by black dashed lines with distances labeled in Å.

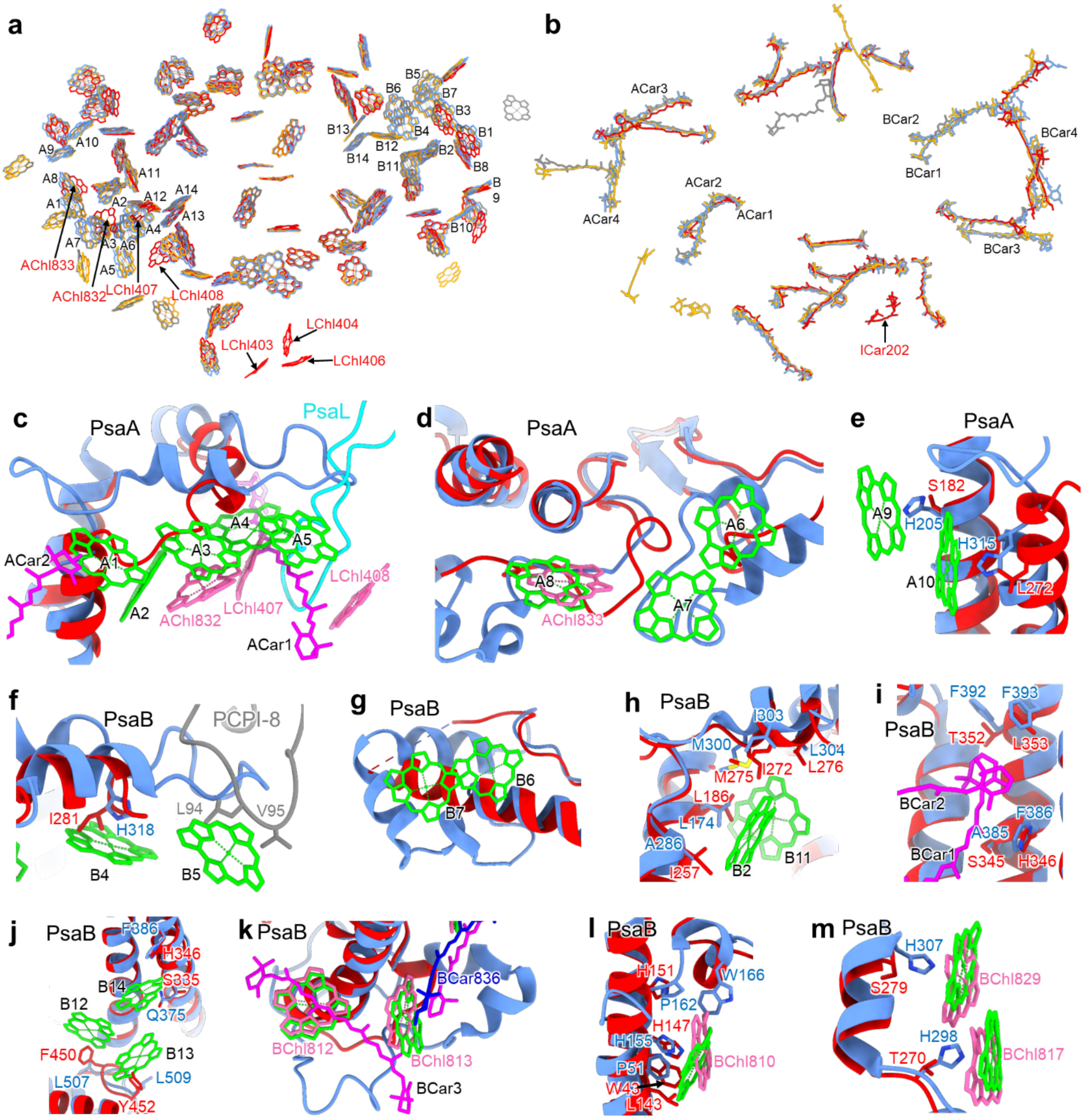

Supplementary Fig. 20. Comparation of pigments arrangements in *Symbiodinium* PSI core with those in red algae (PDB: 7Y5E), cryptophyte (PDB: 7Y7B) and diatom (PDB: 6LY5). a, Superposition of the Chl sites in *Symbiodinium* PSI core (red) with those in red algae (gray), cryptophyte (orange) and diatom (blue) PSI core. Fourteen conserved Chl sites in PsaA subunits of red algae, cryptophyte and diatom are labeled as A1-A14, and the corresponding conserved sites in PsaB are labeled as B1-B14, among which A1-A10, B2, B4-B7, and B10-B14 are absent in *Symbiodinium* PSI core. The previously unidentified Chl sites in *Symbiodinium* PSI core are indicated by solid arrows. **b**, Superposition of the Car sites in *Symbiodinium* (Sy) PSI core (red) with those in red algae (gray), cryptophyte (orange) and diatom (blue). Four conserved Car sites of PsaA subunits are labeled as ACar1-ACar4, and the corresponding conserved sites in PsaB are labeled as BCar1-BCar4, among which ACar1, ACar4, and BCar1-BCar4 are absent in *Symbiodinium* PSI core. The previously unidentified Car site in *Symbiodinium* PSI core is indicated by solid arrow. **c-m**, Detailed view of the comparations of the pigment associations of PsaA subunits (**c-e**) and PsaB subunits (**f-m**) of *Symbiodinium* (red) and diatom (blue). Chls and Cars in *Symbiodinium*, and Chls and Cars in diatom are colored in pink, blue, green and magenta respectively.

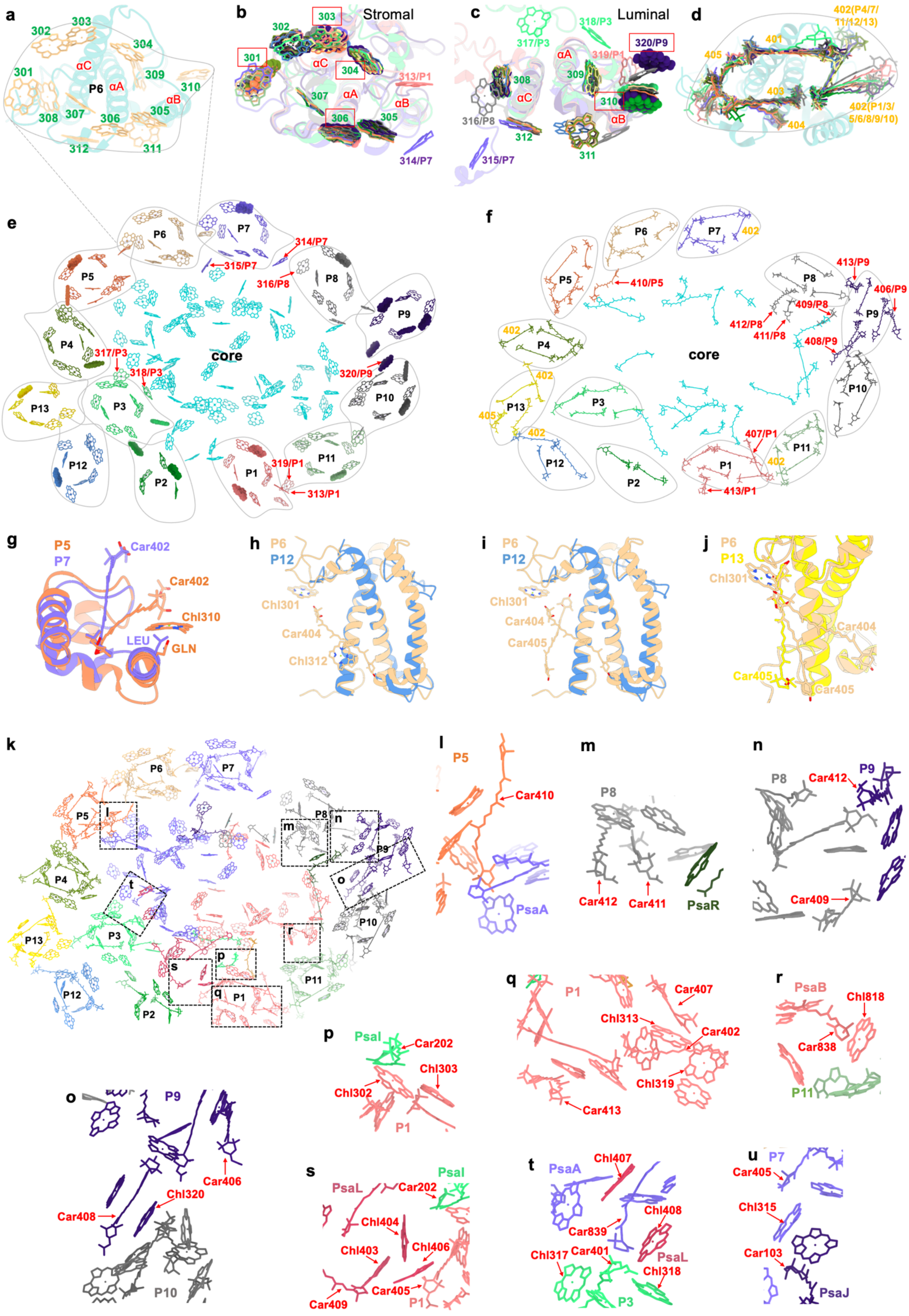

a
b
Stromal
c
Luminal
d
e
core
f
core
g
h
i
j
k
l
m
n
o
p
q
r
s
t
u

Supplementary Fig. 21. Pigments arrangements in AcpPCIs. a, Typical Chl sites in AcpPCI-6 (P6) viewed from the stromal side. Its 12 Chl sites are conserved in most of the red algal LHCRs, Lhcr-type cryptophyte ACPIs and Lhcr-type diatom FCPIs. **b-c**, The Chl sites in AcpPCIs at stromal layer (**b**) and lumenal layer (**c**) viewed from the stromal side. The sites that could bind Chl *c* are indicated by red boxes. Chl *c* is shown in sphere. **d**, The five Car sites conserved in Lhcr-type AcpPCI-4/5/6/10/11. They are also conserved in red algal LHCRs, Lhcr-type cryptophyte ACPIs and Lhcr-type diatom FCPIs. **e**, Arrangement of all Chls in the PSI–AcpPCI. The Chls of each AcpPCI are circled by gray line. The core Chls are colored in cyan. The unique Chl sites are labeled. Chls 313/314/316/319/320 are situated at the interfaces between AcpPCIs, while Chl 315/317/318 are positioned at the interfaces between AcpPCIs and the PSI core. P1-P13 represent AcpPCI-1 to AcpPCI-13. **f**, Arrangement of all Cars in the PSI–AcpPCI. The Cars of each AcpPCI are circled by gray line. The core Chls are colored in cyan. The unique Car sites are labeled. P1-P13 represent AcpPCI-1 to AcpPCI-13. **g**, Comparation of the binding positions of Car402 in AcpPCI-5 (P5) and Car402 in AcpPCI-7 (P7). **h-i**, The locations of Car404, Car405, Chl301 and Chl312 in AcpPCI-6 (P6). These four pigments are absent in AcpPCI-12 (P12). **j**, Comparation of the binding positions of Car405 in AcpPCI-13 (P13) and Car405 in AcpPCI-6 (P6). The lumenal heads of Car 402 in AcpPCI-4/7/11/12/13 exhibit a shift away from the Chl 310 site, which is absent in these AcpPCIs due to the absence of its ligand, Gln (**d-g**). This suggests that Chl 310 may influence the binding of Car 402. Car 404 is not present in AcpPCI-1/2/3/8/9/12/13, nor are adjacent Chls 301 and 312 (**h**). This indicates that the presence of Chls 301 and 312 is important for stabilizing Car 404. The binding sites of Car 405 in AcpPCI-2/8/9/12/13 are not adjacent to other AcpPCIs (**f**). This structural characteristic, coupled with the absence of Chl 301 and Car 404, which are in close proximity to Car 405, may contribute to the dissociation of Car 405 in these AcpPCIs. However, in AcpPCI-13, Car 405 exhibits a shift compared to the other Cars 405 molecules (**i-j**). **k-u**, Potential energy quenching sites in PSI–AcpPCI. Squared areas in **k** are enlarged in **l-u**. P1-P13 represent the AcpPCI-1 to AcpPCI-13 subunits.

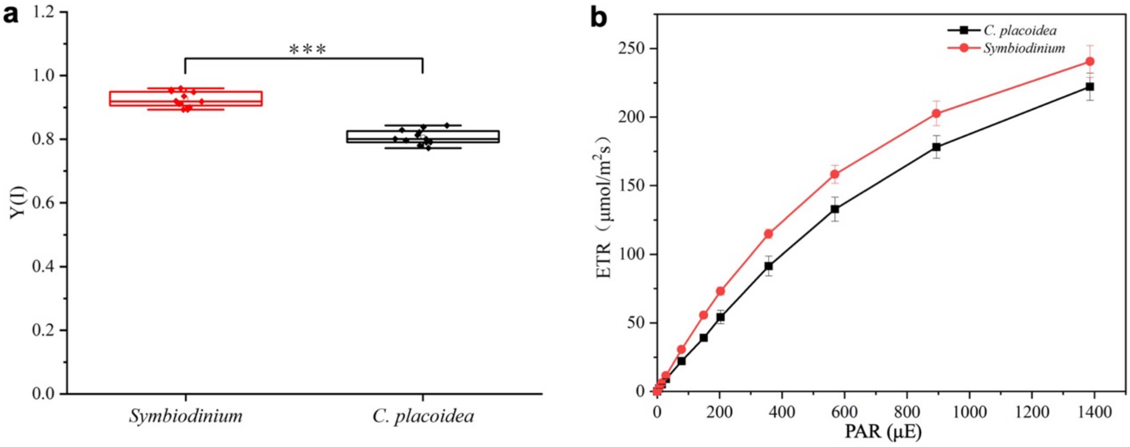

Supplementary Fig. 22. P700 redox kinetics of PSI–LHCI of *Symbiodinium* and the cryptophytic alga *Chroomonas placoidea*. a, Box plots of the PSI photochemical efficiencies of *Symbiodinium* and *C. placoidea*. Box plots display the median (line), the average (open square), the interquartile range (box) and the whiskers (extending 1.5 times the interquartile range). $P = 2.65 \times 10^{-11}$, two-tailed Student's t test, *** $P < 0.005$. **b**, Electron transport rates of *Symbiodinium* and *C. placoidea* PSI–LHCI under different light intensities. Data are means ± SD from three biological repeats. Source data for Supplementary Figs. 22a and 22b are provided.

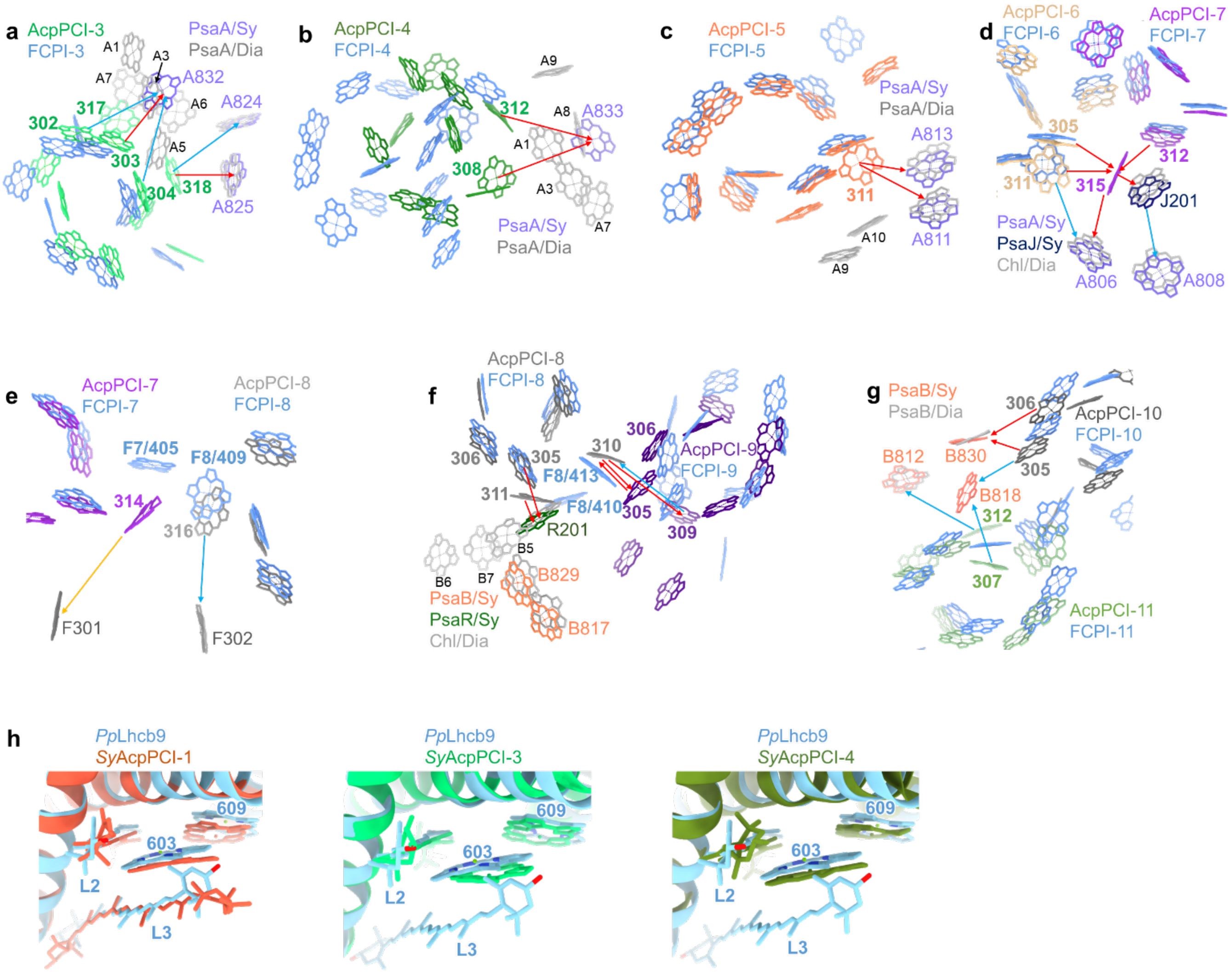

Supplementary Fig. 23. Energy transfer from AcpPCIs to the PSI core in *Symbiodinium* (Sy) PSI–AcpPCI and comparations with those of diatom (Dia) PSI–FCPI (PDB: 6LY5) and moss *Physcomitrium patens* PSI-LHCI (PDB: 8HTU). a-g, Comparations of the arrangements of Chls in AcpPCIs and Chls of PSI core at the interface of AcpPCIs and PSI core with those of diatom PSI-FCPI. Energy transfer pathways from AcpPCIs (**a**: AcpPCI-3, **b**: AcpPCI-4, **c**: AcpPCI-5, **d**: AcpPCI-6/7, **e**: AcpPCI-7/8, **f**: AcpPCI-8/9, **g**: AcpPCI-10) to PSI core are indicated by arrows (Red arrows: faster than 2 ps; blue arrows: between 2 ps and 5 ps; orange arrows: between 5 ps and 10 ps). Chls in diatom FCPIs and PSI core are colored blue and light gray respectively. A1/A3/A5-A10 and B5-B7 are the diatom PSI core Chls which are absent in *Symbiodinium* PSI core. **h**, Comparison of the arrangement of the Chl pair 603/609 and carotenoids L2/L3 in Lhcb9 of moss *Physcomitrium patens* (*Pp*) (PDB: 8HTU) with those in *Symbiodinium* (*Sy*) AcpPCI-1, AcpPCI-3, and AcpPCI-4. The Chl pair 603/609 in Lhcb9 corresponds to the Chl pair 305/306 in AcpPCIs.

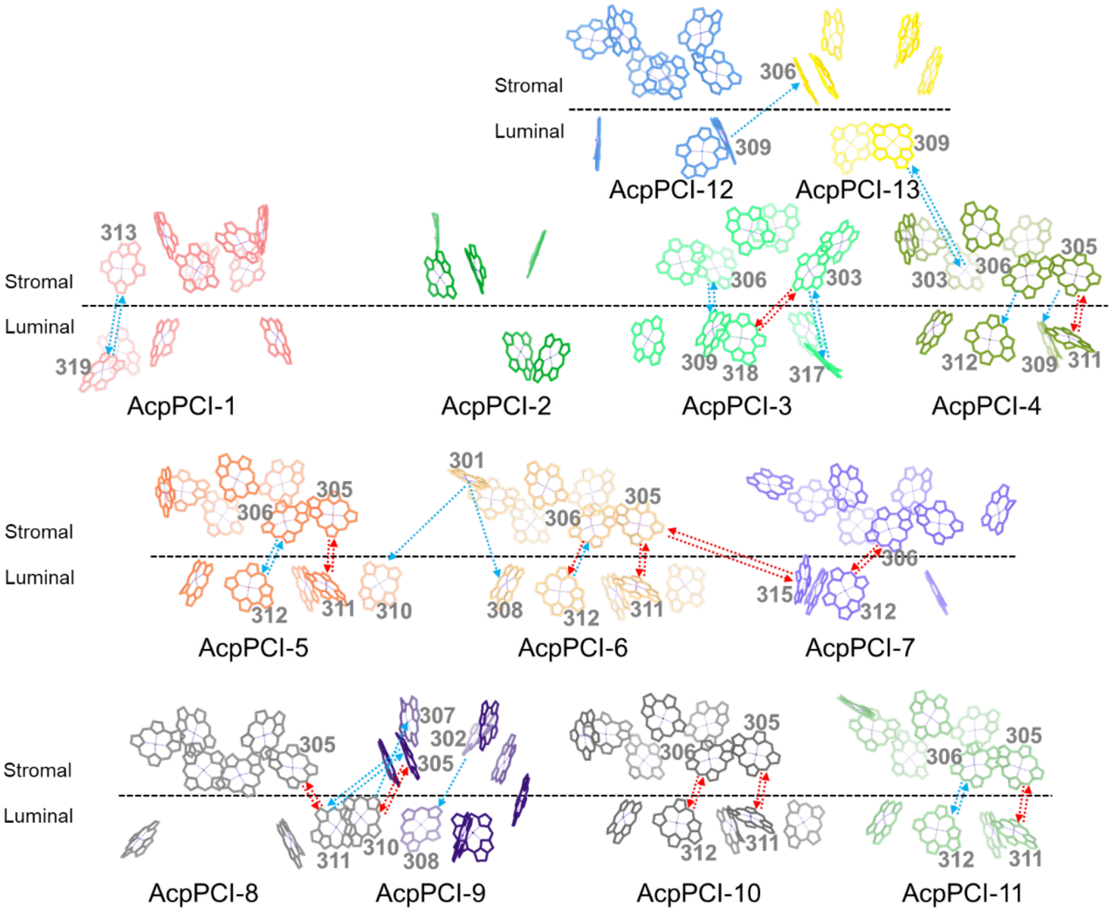

Supplementary Fig. 24. Energy transfer pathways between the stromal and lumenal Chl layer. Side view of Chl arrangement in all AcpPCIs. Black dotted line divides the Chls into the stromal and lumenal layers. Efficient energy transfer pathways between the two layers are indicated by dashed arrows (Red arrows: faster than 2 ps; blue arrows: between 2 ps and 5 ps).

Supplementary Table 1. Cryo-EM data collection, refinement, and validation statistics.

	**PSI–AcpPCI (EMDB-36366; PDB-8JJR)**
**Data Collection and Processing**	
Voltage (kV)	300
Electron exposure (e- /Å2)	60
Defocus range (um)	-1.2~-2.2
Pixel size (Å)	0.53
Symmetry imposed	C1
Initial particle images (no.)	1,123,901
Final particle images (no.)	161,863
Map resolution (Å)	2.80
FSC threshold	0.143
**Refinement**	
Initial model used (PDB code)	6LY5
Model resolution (Å)	2.80
FSC threshold	0.143
Map sharpening *B* factor (Å^2)	-85.5
Model composition	
Non-hydrogen atoms	55973
Protein residues	5073
Ligands	323
B factors (Å^2)	
Protein	32.92
Ligand	30.90
R.m.s. deviations	
Bond lengths (Å)	0.009
Bond angles (°)	1.334
Validation	
MolProbity score	1.66
Clashscore	8.49
Poor rotamers (%)	0.12
Ramachandran plot	
Favored (%)	96.77
Allowed (%)	3.19
Disallowed (%)	0.04

Supplementary Table 2. Cofactors in each subunit of the *Symbiodinium* PSI–AcpPCI structure.

Subunit	Traced residues	Chls	Cars	Lipids	Others
PsaA	687 (1-687)	36 a	3 β-Car	1DGDG, 1 PG	1 PQN
PsaB	666 (4-669)	32 a	3 β-Car, 1 Ddx	1 SQDG, 1 MGDG, 1 PG	1 PQN, 1SF4
PsaC	86 (76-161)				2 SF4
PsaD	219 (77-295)				
PsaE	73 (49-121)				
PsaF	169 (111-279)	3 a	1 β-Car	1 DGDG	
PsaI	120 (60-179)		1 β-Car, 1Ddx		
PsaJ	99 (43-141)	1 a	1 β-Car, 1Dx	1 PG	
PsaL	253 (108-360)	8 a	3 β-Car	1 DGDG, 1 MGDG	
PsaM	79 (64-142)		1 β-Car		
PsaR	131 (22-152)	1 a	2 Ddx		
PsaT	79 (40-118)				
PsaU	129 (94-223)				
AcpPCI-1	185 (75-259)	9 a, 2 c	3 Ddx, 2 Dx, 1 Per	1 PG	
AcpPCI-2	121 (1-121)	4 a, 2 c	1 Ddx, 1 Per		
AcpPCI-3	221 (26-246)	10 a, 1 c	2 Ddx, 1 Dx, 1 Per		
AcpPCI-4	173 (28-200)	10 a, 1 c	4 Ddx, 1 Dx	1 PG	
AcpPCI-5	184 (59-242)	10 a, 2 c	5 Ddx, 1 Dx	1 SQDG	
AcpPCI-6	177 (1-177)	12 a	4 Ddx, 1 Dx	1 PG, 2 MGDG	
AcpPCI-7	194 (58-251)	11 a, 1 c	3 Ddx, 2 Dx	1 SQDG	
AcpPCI-8	225 (62-286)	9 a, 1 c	3 Ddx, 1 Dx, 2 Per	1 MGDG	
AcpPCI-9	173 (31-203)	5 a, 5 c	1 Ddx, 1 Dx, 4 Per	1 PG	
AcpPCI-10	192 (80-271)	10 a, 2 c	3 Ddx, 2 Dx		
AcpPCI-11	192 (1-192)	10 a, 1 c	3 Ddx, 2 Dx	1 MGDG	
AcpPCI-12	140 (60-199)	8 a, 1 c	2 Ddx, 1 Dx		
AcpPCI-13	154 (73-226)	7 a, 1 c	3 Ddx, 1 Per	1 MGDG, 1 DGDG	
PSI–AcpPCI		196 a, 20 c	41 Ddx, 13 β-Car, 16 Dx, 10 Per	7 PG, 7 MGDG, 4 DGDG, 3 SQDG	2 PQN, 3 SF4

Ddx, diadinoxanthin; *β*-Car, *β*-carotene; Dx, dinoxanthin; Per, peridinin; DGDG, digalactosyldiacyl glycerol; MGDG, monogalactosyldiacyl glycerol; PG, phosphatidyl glycerol; SQDG, sulfoquinovosyldiacyl glycerol; PQN, phylloquinone; SF4, sulphur–iron cluster.

Supplementary Table 3. Comparation of the protein subunits in PSI–LHCI of *Symbiodinium*, cyanobacteria[1], red algae[2, 3], cryptophyte[4], diatom[5], green algae[6-10], moss[11, 12, 13, 14] and plants[15, 16]. √: presence of the subunit.

**Subunit**	***Symbiodinium***	**Cyanobacteria**	**Red algae**	**Cryptophyte**	**Diatom**	**Green algae**	**Moss**	**Plants**
PsaA	√	√	√	√	√	√	√	√
PsaB	√	√	√	√	√	√	√	√
PsaC	√	√	√	√	√	√	√	√
PsaD	√	√	√	√	√	√	√	√
PsaE	√	√	√	√	√	√	√	√
PsaF	√	√	√	√	√	√	√	√
PsaG						√	√	√
PsaH						√	√	√
PsaI	√	√	√	√	√	√	√	√
PsaJ	√	√	√	√	√	√	√	√
PsaK		√	√	√		√	√	√
PsaL	√	√	√	√	√	√	√	√
PsaM	√	√	√	√	√	√	√	
PsaN						√		√
PsaO			√	√		√	√	√
PsaR	√		√	√	√			
PsaS					√			
PsaX		√						
PsaT	√							
PsaU	√							
Unk1				√				
ACPI-S				√				
LHCI *n*	13	0	3, 5 or 8	11 or 14	24	6, 8, 9 or 10	4 or 8	4

Supplementary Table 4. Binding sites of pigments in AcpPCI subunits. Chl *c* is colored in blue in contrast to Chl *a* in black.

Sites	AcpPCI -1	AcpPCI -2	AcpPCI -3	AcpPCI -4	AcpPCI -5	AcpPCI -6	AcpPCI -7	AcpPCI -8	AcpPCI -9	AcpPCI -10	AcpPCI -11	AcpPCI -12	AcpPCI -13
301				Chl *c*	Chl *c*	Chl *a*	Chl *a*			Chl *c*	Chl *a*		
302	Chl *a*		Chl *a*	Chl *a*	Chl *a*	Chl *a*	Chl *a*	Chl *a*	Chl *a*	Chl *a*	Chl *a*	Chl *a*	Chl *a*
303	Chl *c*		Chl *a*	Chl *a*	Chl *c*	Chl *a*	Chl *c*	Chl *c*	Chl *c*	Chl *c*	Chl *c*	Chl *c*	Chl *c*
304	Chl *a*	Chl *a*	Chl *a*	Chl *a*	Chl *a*	Chl *a*	Chl *a*	Chl *a*	Chl *c*	Chl *a*	Chl *a*	Chl *a*	Chl *a*
305	Chl *a*	Chl *a*	Chl *a*	Chl *a*	Chl *a*	Chl *a*	Chl *a*	Chl *a*	Chl *a*	Chl *a*	Chl *a*	Chl *a*	Chl *a*
306	Chl *c*	Chl *c*	Chl *a*	Chl *a*	Chl *a*	Chl *a*	Chl *a*	Chl *a*	Chl *c*	Chl *a*	Chl *a*	Chl *a*	Chl *a*
307	Chl *a*	Chl *a*	Chl *a*	Chl *a*	Chl *a*	Chl *a*	Chl *a*	Chl *a*	Chl *a*	Chl *a*	Chl *a*	Chl *a*	Chl *a*
308	Chl *a*		Chl *a*	Chl *a*	Chl *a*	Chl *a*	Chl *a*		Chl *a*	Chl *a*	Chl *a*	Chl *a*	Chl *a*
309	Chl *a*	Chl *a*	Chl *a*	Chl *a*	Chl *a*	Chl *a*	Chl *a*	Chl *a*	Chl *a*	Chl *a*	Chl *a*	Chl *a*	Chl *a*
310	Chl *a*	Chl *c*	Chl *c*		Chl *a*	Chl *a*		Chl *a*	Chl *c*	Chl *a*			
311				Chl *a*	Chl *a*	Chl *a*		Chl *a*		Chl *a*	Chl *a*	Chl *a*	
312				Chl *a*	Chl *a*	Chl *a*	Chl *a*			Chl *a*	Chl *a*		
313	Chl *a*												
314							Chl *a*						
315							Chl *a*						
316								Chl *a*					
317			Chl *a*										
318			Chl *a*										
319	Chl *a*												
320									Chl *c*				
401	Dx		Dx	Ddx	Ddx	Ddx	Dx	Ddx	Per	Dx	Dx	Ddx	Ddx
402	Ddx	Per	Ddx	Dx	Dx	Dx	Dx	Dx	Per	Dx	Dx	Dx	Ddx
403	Ddx	Ddx	Ddx	Ddx	Ddx	Ddx	Ddx	Ddx	Ddx	Ddx	Ddx	Ddx	Ddx
404				Ddx	Ddx	Ddx	Ddx			Ddx	Ddx		
405	Per		Per	Ddx	Ddx	Ddx	Ddx			Ddx	Ddx		Per
406									Per				
407	Ddx												
408									Per				
409								Ddx					
410					Ddx								
411								Per					
412								Per					
413	Dx								Dx				

Supplementary Table 5. Calculated EET time constant between AcpPCIs and the PSI core based on the generalised Förster theory. Letters of P1 to P13 represent AcpPCI-1 to AcpPCI-13.

EET route	EET time constant (ps)
P1→PSI core	1.1
P2→PSI core	10.4
P2→P3→PSI core	16.6
P13→P12→P3→PSI core	12.0
P13→P3→PSI core	11.8
P13→P4→P3→PSI core	12.7
P4→P3→PSI core	5.4
P5→PSI core	3.8
P6→P7→PSI core	2.1
P9→P8→PSI core	9.7
P11→P10→PSI core	3.0
P11→PSI core	5.3

Supplementary Table 6. Possible EET pathways from AcpPCIs to the PSI core based on the Förster theory. Letters of P1 to P13 represent AcpPCI-1 to AcpPCI-13. Red text: faster than 2 ps; blue text: between 2 ps and 5 ps; orange text: between 5 ps and 10 ps.

Possible pathway	Pigments
P1→PSI core	P1: Chl 307→PsaL: Chl 406
	P1: Chl 302/308→PsaL: Chl 404/403
P2→PSI core	P2: Chl 309/305→PsaL: Chl 403/405
P2→P3→PSI core	P2: Chl 305→P3: Chl 305
	P3: Chl 303/318→PsaA: Chl 832/825
	P3: Chl 302/304→PsaA: Chl 832/824
P13→P12→P3→PSI core	P13: Chl 305/306/307→P12: Chl 305/309/304
	P12: Chl 305→P3: Chl 306
	P3: Chl 303/318→PsaA: Chl 832/825
	P3: Chl 302/304→PsaA: Chl 832/824
P13→P3→PSI core	P13: Chl 305→P3: Chl 306
	P3: Chl 303/318→PsaA: Chl 832/825
	P3: Chl 302/304→PsaA: Chl 832/824
P13→P4→P3→PSI core	P13: Chl 304/309→P4: Chl 303
	P4: Chl 302→P3: Chl 302
	P3: Chl 303/318→PsaA: Chl 832/825
	P3: Chl 302/304→PsaA: Chl 832/824
P4→PSI core	P4: Chl 308/312→PsaA: Chl 833
P5→PSI core	P5: Chl 311→PsaA: Chl 813/811
P6→PSI core	P6: Chl 311/312→PsaA: Chl 806/813
P6→P7→PSI core	P6: Chl 304→P7: Chl 301
	P6: Chl 310→P7: Chl 308/312
	P6: Chl 305/311→P7: Chl 315
	P7: Chl 315→PsaJ: Chl 201
	P7: Chl 315→PsaA: Chl 806
P9→P8→PSI core	P9: Chl 309/305→P8: Chl 310/311
	P9: Chl 305→P8: Chl 310
	P8: Chl 305/311→PsaR: Chl 201
P9→PSI core	P9: Chl 305→PsaR: Chl 201
P11→P10→PSI core	P11: Chl 308/312/301→P10: Chl 310/304
	P10: Chl 305/306→PsaB: Chl 830
	P10: Chl 305→PsaB: Chl 818
P11→PSI core	P11: Chl 307/312→PsaB: Chl 818/812

Supplementary Table 7. Possible EET pathways between AcpPCIs based on the Förster theory. Letters of P1 to P13 represent AcpPCI-1 to AcpPCI-13. Red text: faster than 2 ps; blue text: between 2 ps and 5 ps; orange text: between 5 ps and 10 ps.

Possible pathway	Pigments
P2↔P3	P2: Chl 305→P3: Chl 305
	P3: Chl 305→P2: Chl 305
P3↔P4	P3: Chl 302↔P4: Chl 302
P4↔P5	P4: Chl 312↔P5: Chl 312
	P4: Chl 312→P5: Chl 308
	P5: Chl 308→P4: Chl 312
	P5: Chl 312→P4: Chl 311
P5↔P6	P5: Chl 304→P6: Chl 301
	P6: Chl 301→P5: Chl 304
	P5: Chl 310→P6: Chl 312
	P6: Chl 312→P5: Chl 310
	P5: Chl 310↔P6: Chl 308
	P6: Chl 301→P5: Chl 310
	P6: Chl 312→P5: Chl 311
P6↔P7	P6: Chl 305/311↔P7: Chl 315
	P6: Chl 310→P7: Chl 308/312
	P7: Chl 308/312→P6: Chl 310
	P6: Chl 304→P7: Chl 301
	P7: Chl 301→P6: Chl 304
P8↔P9	P8: Chl 310↔P9: Chl 305
	P8: Chl 311↔P9: Chl 305
	P8: Chl 310→P9: Chl 307
	P8: Chl 310→P9: Chl 309
	P9: Chl 309→P8: Chl 310
P9↔P10	P9: Chl 320→P10: Chl 312
P10↔P11	P10: Chl 310↔P11: Chl 312/308
	P10: Chl 304→P11: Chl 301
	P11: Chl 301→P10: Chl 304
P11↔P1	P11: Chl 309↔P1: Chl 319
P12↔P3	P12: Chl 305↔P3: Chl 306
	P12: Chl 306↔P3: Chl 306
P12↔P13	P12: Chl 305→P13: Chl 305
	P12: Chl 309→P13: Chl 306
	P13: Chl 305→P12: Chl 305/306
	P13: Chl 306→P12: Chl 304/309
	P13: Chl 307→P12: Chl 304
P13↔P4	P13: Chl 309↔P4: Chl 303
	P13: Chl 304→P4: Chl 303
	P4: Chl 303→P13: Chl 304

Supplementary Table 8. Primers used in this work

Primer	Gene sequence
Euk328f	5'-ACCTGGTTGATCCTGCCAG-3'
Euk329r	5'-TGATCCTTCYGCAGGTTCAC-3'
18S.comm.F1	5’-GTCGTAACAAGGTTTCYGTAGGTG-3’
28S.sym.R1	5’-GTTYDCTTGYYTGACTTCATGCTA-3’

Supplementary References

1. Jordan, P. et al. Three-dimensional structure of cyanobacterial photosystem I at 2.5 A resolution. *Nature* **411**, 909-917 (2001).
2. Pi, X. et al. Unique organization of photosystem I-light-harvesting supercomplex revealed by cryo-EM from a red alga. *Proc Natl Acad Sci U S A* **115**, 4423-4428 (2018).
3. You, X. et al. In situ structure of the red algal phycobilisome-PSII-PSI-LHC megacomplex. *Nature* **616**, 199-206 (2023).
4. Zhao, L.S. et al. Structural basis and evolution of the photosystem I-light-harvesting supercomplex of cryptophyte algae. *Plant Cell* (2023).
5. Xu, C. et al. Structural basis for energy transfer in a huge diatom PSI-FCPI supercomplex. *Nat Commun* **11**, 5081 (2020).
6. Qin, X. et al. Structure of a green algal photosystem I in complex with a large number of light-harvesting complex I subunits. *Nat Plants* **5**, 263-272 (2019).
7. Su, X. et al. Antenna arrangement and energy transfer pathways of a green algal photosystem-I-LHCI supercomplex. *Nat Plants* **5**, 273-281 (2019).
8. Caspy, I. et al. Structure and energy transfer pathways of the Dunaliella Salina photosystem I supercomplex. *Biochim Biophys Acta Bioenerg* **1861**, 148253 (2020).
9. Ishii, A. et al. The photosystem I supercomplex from a primordial green alga Ostreococcus tauri harbors three light-harvesting complex trimers. *Elife* **12** (2023).
10. Naschberger, A. et al. Algal photosystem I dimer and high-resolution model of PSI-plastocyanin complex. *Nat Plants* **8**, 1191-1201 (2022a).
11. Yan, Q. et al. Antenna arrangement and energy-transfer pathways of PSI–LHCI from the moss Physcomitrella patens. *Cell Discovery* **7**, 10 (2021).
12. Gorski C. et al. The structure of the Physcomitrium patens photosystem I reveals a unique Lhca2 paralogue replacing Lhca4. *Nat Plants* **8**, 307–316 (2022).
13. Zhang S. et al. Structural insights into a unique PSI–LHCI–LHCII–Lhcb9 supercomplex from moss Physcomitrium patens. *Nat Plants* **9**, 832–846 (2023).
14. Sun, H.Y., Shang, H., Pan, X.W. & Li, M. Structural insights into the assembly and energy transfer of the Lhcb9-dependent photosystem I from moss. *Nat Plants* **9**, 1347-1358 (2023).
15. Qin, X., Suga, M., Kuang, T. & Shen, J.R. Photosynthesis. Structural basis for energy transfer pathways in the plant PSI-LHCI supercomplex. *Science* **348**, 989-995 (2015).
16. Pan, X. et al. Structure of the maize photosystem I supercomplex with light-harvesting complexes I and II. *Science* **360**, 1109-1113 (2018).
