# Supplementary material for: Architecture of symbiotic dinoflagellate photosystem I–light-harvesting supercomplex in Symbiodinium
Source: Nat Commun. 2024 Mar 16;15:2392. doi: 10.1038/s41467-024-46791-x (PMC10944487; doi:10.1038/s41467-024-46791-x)
Supplement: Supplementary file 3 — Description of Additional Supplementary Files [file 41467_2024_46791_MOESM3_ESM.pdf]

Description of Additional Supplementary Files

File Name: Supplementary Data 1
Description: Mass spectrometry analysis of proteins determined in SDS-PAGE in Supplementary Figure 1d.
