# Supplementary material for: Architecture of symbiotic dinoflagellate photosystem I–light-harvesting supercomplex in Symbiodinium
Source: Nat Commun. 2024 Mar 16;15:2392. doi: 10.1038/s41467-024-46791-x (PMC10944487; doi:10.1038/s41467-024-46791-x)
Supplement: Supplementary file 5 — Reporting Summary [file 41467_2024_46791_MOESM5_ESM.pdf]

nature portfolio

Corresponding author(s): Jun Gao, Lu-Ning Liu, Yu-Zhong Zhang

Last updated by author(s): Mar 8, 2024

# Reporting Summary

Nature Portfolio wishes to improve the reproducibility of the work that we publish. This form provides structure for consistency and transparency in reporting. For further information on Nature Portfolio policies, see our Editorial Policies and the Editorial Policy Checklist.

## Statistics

For all statistical analyses, confirm that the following items are present in the figure legend, table legend, main text, or Methods section.

| n/a | Confirmed | |
|---|---|---|
| ☐ | ☒ | The exact sample size (*n*) for each experimental group/condition, given as a discrete number and unit of measurement |
| ☐ | ☒ | A statement on whether measurements were taken from distinct samples or whether the same sample was measured repeatedly |
| ☐ | ☒ | The statistical test(s) used AND whether they are one- or two-sided<br>*Only common tests should be described solely by name; describe more complex techniques in the Methods section.* |
| ☒ | ☐ | A description of all covariates tested |
| ☒ | ☐ | A description of any assumptions or corrections, such as tests of normality and adjustment for multiple comparisons |
| ☐ | ☒ | A full description of the statistical parameters including central tendency (e.g. means) or other basic estimates (e.g. regression coefficient) AND variation (e.g. standard deviation) or associated estimates of uncertainty (e.g. confidence intervals) |
| ☐ | ☒ | For null hypothesis testing, the test statistic (e.g. $F$, $t$, $r$) with confidence intervals, effect sizes, degrees of freedom and $P$ value noted<br>*Give P values as exact values whenever suitable.* |
| ☒ | ☐ | For Bayesian analysis, information on the choice of priors and Markov chain Monte Carlo settings |
| ☒ | ☐ | For hierarchical and complex designs, identification of the appropriate level for tests and full reporting of outcomes |
| ☒ | ☐ | Estimates of effect sizes (e.g. Cohen's *d*, Pearson's *r*), indicating how they were calculated |

*Our web collection on statistics for biologists contains articles on many of the points above.*

## Software and code

Policy information about availability of computer code

| | |
|---|---|
| Data collection | Cryo-EM Data collection were performed on a 300 kV Titan Krios G3i microscope (Thermo Fisher Scientific) equipped with a K3 BioQuantum direct electron detector (Gatan Inc.). Movie stacks were recorded using EPU (Thermo Fisher Scientific). Absorption spectra were measured using a Shimadzu UV–Vis 1990 spectrophotometer. Pigment composition was analyzed by LC-20AD high performance liquid chromatograph (Shimadzu, Janpan). P700 oxidation kinetics were recorded using a pulse amplitude modulated fluorometer Dual-PAM-100 (Walz, Germany). The cDNA library preparations were sequenced on an Illumina HiSeq 2000 platform. |
| Data analysis | cryoSPARC 3.3.1, UCSF ChimeraX, CCP4 7.0, WinCOOT 0.9.8.1, PHENIX 1.20, ESPript 3.0, Trinity 2.5.1, CLC Sequence Viewer 8, MEGA X, Gaussian16 software, Custom python scripts deposited in GitHub [https://doi.org/10.5281/zenodo.10791187]. |

For manuscripts utilizing custom algorithms or software that are central to the research but not yet described in published literature, software must be made available to editors and reviewers. We strongly encourage code deposition in a community repository (e.g. GitHub). See the Nature Portfolio guidelines for submitting code & software for further information.

## Data

Policy information about availability of data

All manuscripts must include a data availability statement. This statement should provide the following information, where applicable:

- Accession codes, unique identifiers, or web links for publicly available datasets
- A description of any restrictions on data availability
- For clinical datasets or third party data, please ensure that the statement adheres to our policy

The cryo-EM map and atomic coordinates generated in this study have been deposited in the Protein Data Bank and the Electron Microscopy Data Bank under the accession numbers of 8JJR [https://www.rcsb.org/structure/unreleased/8JJR] and EMD-36366 [https://www.ebi.ac.uk/emdb/EMD-36366], respectively. The atomic coordinates data used in this study are available in the Protein Data Bank database under accession code 6LY5 [https://www.rcsb.org/structure/6LY5], 7Y5E [https://www.rcsb.org/structure/7Y5E], and 7Y7B [https://www.rcsb.org/structure/7Y7B]. The 18S ribosomal RNA and internal transcribed spacer (ITS) gene sequences of Symbiodinium sp. GY-H50 have been deposited to the National Center for Biotechnology Information database with the accession numbers PP191135 [https://www.ncbi.nlm.nih.gov/nuccore/PP191135] and PP191136 [https://www.ncbi.nlm.nih.gov/nuccore/PP191136]. The ribosomal RNA gene sequences of PsaT and PsaU have been deposited to the National Center for Biotechnology Information database with the accession numbers PP196340 [https://www.ncbi.nlm.nih.gov/nuccore/PP196340] and PP196339 [https://www.ncbi.nlm.nih.gov/nuccore/PP196339], respectively. Source data for Supplementary Figs. 1b, 1c, 1e, and Supplementary Figs. 22a, 22b are provided in the Source Data file.

## Research involving human participants, their data, or biological material

Policy information about studies with human participants or human data. See also policy information about sex, gender (identity/presentation), and sexual orientation and race, ethnicity and racism.

| | |
|---|---|
| Reporting on sex and gender | N/A |
| Reporting on race, ethnicity, or other socially relevant groupings | N/A |
| Population characteristics | N/A |
| Recruitment | N/A |
| Ethics oversight | N/A |

Note that full information on the approval of the study protocol must also be provided in the manuscript.

# Field-specific reporting

Please select the one below that is the best fit for your research. If you are not sure, read the appropriate sections before making your selection.

☒ Life sciences ☐ Behavioural & social sciences ☐ Ecological, evolutionary & environmental sciences

For a reference copy of the document with all sections, see nature.com/documents/nr-reporting-summary-flat.pdf

# Life sciences study design

All studies must disclose on these points even when the disclosure is negative.

| | |
|---|---|
| Sample size | Five independent samples were measured for the absorption spectra and P700 oxidation kinetics. Pigments of Three independent samples were analyzed by HPLC. Five batches of samples were analyzed by SDS-PAGE. Similar results were obtained from the replicates of each experimental analysis with good biological reproducibility. A total of 6092 movies were recorded. More than280,000 protein particles were picked for further process. Finally, 118,810 protein particles were selected for refinement. During the image processing, only the movies and particles with high quality and resolution were used for map construction. According to our previous knowledge on data processing and the data processing of published structures of photosystems, 118,810 protein particles with high quality and resolution are sufficient for building a map of photosystem with atomic resolution. No statistical methods were used to predetermine sample size. |
| Data exclusions | A fraction of the low quality and low resolution cryo-EM movies and particles were discarded. |
| Replication | The purification and characterization of PSI-AcpPCI (SDS-PAGE, absorption spectrum, pigment analysis, and P700 oxidation kinetics) have been repeated independently for more than three times. Similar results were obtained from the replicates of each experimental analysis. |
| Randomization | Randomization is not relevant to our study as the goal of this study is to solve the structure of the specific protein supercomplex. |
| Blinding | Blinding is not relevant to our study because we are studying a specific protein complex. There was no existing protein structure to refer to and the data analysis was ab initio. |

# Reporting for specific materials, systems and methods

We require information from authors about some types of materials, experimental systems and methods used in many studies. Here, indicate whether each material, system or method listed is relevant to your study. If you are not sure if a list item applies to your research, read the appropriate section before selecting a response.

## Materials & experimental systems

| n/a | Involved in the study |
| --- | --- |
| ☒ | ☐ Antibodies |
| ☒ | ☐ Eukaryotic cell lines |
| ☒ | ☐ Palaeontology and archaeology |
| ☒ | ☐ Animals and other organisms |
| ☒ | ☐ Clinical data |
| ☒ | ☐ Dual use research of concern |
| ☒ | ☐ Plants |

## Methods

| n/a | Involved in the study |
| --- | --- |
| ☒ | ☐ ChIP-seq |
| ☒ | ☐ Flow cytometry |
| ☒ | ☐ MRI-based neuroimaging |

## Plants

| | |
| --- | --- |
| Seed stocks | N/A |
| Novel plant genotypes | N/A |
| Authentication | N/A |
